# Fluorophore-Tagged Poly-Lysine RAFT Agents: Controlled Synthesis of Trackable Cell-Penetrating Peptide–Polymers

**DOI:** 10.1021/acsmacrolett.3c00460

**Published:** 2023-09-11

**Authors:** Paige A. Shaw, Maxime Klausen, Annamaria Lilienkampf, Mark Bradley

**Affiliations:** † EaStCHEM School of Chemistry, University of Edinburgh, David Brewster Road, EH9 3FJ Edinburgh, U.K.; ‡ Precision Healthcare University Research Institute, Queen Mary University of London, 65-67 New Road, E1 1HH London, U.K.

## Abstract

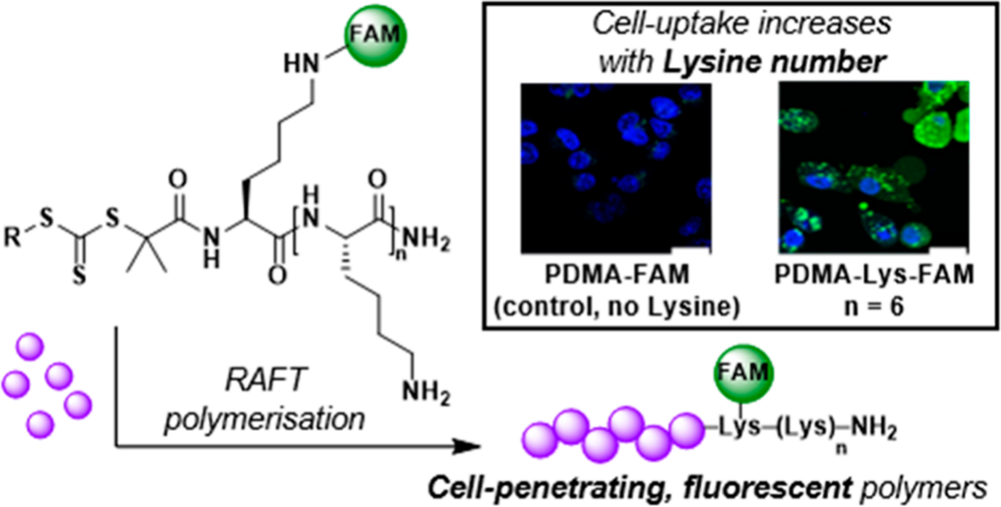

The conjugation of a fluorophore and a variety of cell-penetrating peptides onto a RAFT agent allowed for the synthesis of polymers of defined sizes with quantifiable cell-uptake. Each peptide–RAFT agent was used to polymerize acrylamide, acrylate, and styrene monomers to form high or low molecular weight polymers (here 50 or 7.5 kDa) with the peptide having no influence on the RAFT agent’s control. The incorporation of a single fluorophore per polymer chain allowed cellular analysis of the uptake of the size-specific peptide–polymers via flow cytometry and confocal microscopy. The cell-penetrating peptides had a direct effect on the efficiency of polymer uptake for both high and low molecular weight polymers, demonstrating the versatility of the strategy. These “all-in-one”, synthetically accessible RAFT agents allow highly controlled preparation of synthetic peptide–polymer conjugates and subsequent quantification of their delivery into cells.

Polymers are widely used as carriers in formulations to overcome the pharmacological limitations of many therapeutic agents. Nanoencapsulation,^1^ nanoinjection,^2^ and chemical modification^3^ of drugs with biocompatible polymers have been successfully applied to solve challenges with pharmacokinetics and pharmacodynamics, which has led to a variety of FDA-approved polymer-containing therapeutics with improved bioavailability^4^ and circulation time,^5^ or drug-release control.^6^ However, a challenge lies in the development of methods to target the interaction between polymers and biological tissues and also in the accurate quantification of their uptake and activity in cells.

With their combination of two classes of components, peptide–polymer conjugates offer materials with unique attributes for healthcare applications.^7^ The advantages of each building block, synergistically combined, generates materials with high functional group densities due to the polymer chain and selective biological activity arising from the peptide, while retaining their abilities to tune solubility and topology. This allows multidrug loading across the polymer/peptide chain and makes them versatile high-loading carriers for therapeutic and diagnostic applications.^8−10^ In particular, specific peptides with the ability to penetrate through cell membranes are easily synthesized by solid-phase methods.^11^ This includes classical cell-penetrating peptides (CPP) such as Transportan 10,^12^ Penetratin,^13^ and the TAT peptide,^14^ as well as simpler highly cationic structures such as poly-Lysine^15−17^ and poly-Arginine^18^ that are able to assist cellular uptake in similar ways via various mechanisms. One way to incorporate CPPs onto polymer chains is to use peptide-based chain transfer agents for controlled RAFT polymerization.^19−22^ Hentschel et al. developed integrin-binding peptide–polymer hybrids by attaching RAFT agents onto peptides using solid-phase peptide synthesis.^20^ Chen et al. achieved high cell penetration efficiency with hybrid nanoparticles made of the Transportan 10 peptide conjugated to a diblock copolymer (poly[oligo(ethylene glycol) methyl ether acrylate]-*b*-poly(*n*-butyl acrylate) (TP-POEGA-*b*-PBA) fabricated via RAFT polymerization.^21^

To assess the biological performance of these peptide–polymer conjugates, cellular uptake and localization are commonly evaluated by well-known fluorescence-based assays. The postpolymerization conjugation of fluorophores onto end-groups^19,23−26^ and the copolymerization of fluorescent monomers are common strategies allowing the quantification of the cellular uptake of polymers.^26,27^ However, postpolymerization tagging can lead to incomplete functionalization, while incorporation of fluorescent monomers leads to varying numbers of fluorophores per polymer chain, which can result in fluorescence quenching and hinder quantification. Therefore, although routinely used, these visualization strategies can lead to inaccurate quantification. In contrast, the incorporation of a single fluorescent tracker per chain would provide a reliable and reproducible comparison of the polymer’s cellular uptake. We previously developed fluorescein- and BODIPY-based fluorescent RAFT agents that allowed photopolymerization of a variety of acrylate and acrylamide monomers and led to polymer chains containing a single fluorescent molecule per chain.^28^ Here, our aim was to develop an accessible “all-in-one” solution allowing the preparation of peptide–polymer conjugates, usable for the controlled delivery of polymeric cargo into cells, in which cellular uptake and localization can be accurately followed by fluorescence techniques. To achieve this, RAFT agents combining both a fluorophore and CPP moieties were synthesized using convenient solid-phase techniques. These “all-in-one” tools were thus designed to promote the controlled synthesis of peptide–polymer conjugates with improved cell-penetrating properties, while simultaneously ensuring that every polymer chain contains a single fluorophore and allowing for a direct comparison of peptide-activity on polymer cell uptake. These RAFT agents allowed for the controlled polymerization of *N*,*N*-dimethylacrylamide (DMA), 2-hydroxyethyl acrylate (2HEA), and styrene and yielded fluorescently tagged polymer–peptide conjugates. We demonstrated the peptide moiety promoted cellular uptake of polymers with molecular weights of up to 50 kDa. By modifying the cationic strength of the CPP–RAFT agent, we were able to directly compare the resulting increase in the polymers’ cell uptake, with an increase in the cationic strength of the CPP resulting in significantly increased cell uptake responses for both long and short polymers.

The peptides were conjugated to the RAFT agents via the “R group”, known as the activating moiety of the RAFT agent, meaning that the resulting polymer chains would each contain a CPP–fluorophore conjugate as the “end group”. 5,6-Carboxyfluorescein (FAM, λ_Ex/Em_ = 495/517 nm) was chosen as the fluorophore due to its robustness and bright fluorescence, low toxicity, low hydrophobicity at physiological pH, convenient functionalization, and, importantly, absence of interference with the activity of the peptide.^29^ Poly-Lysine chain lengths of 3, 5, or 7 Lysine residues were investigated (with one of the Lysine side chains used for FAM conjugation), as higher charge densities can result in cell toxicity.^30^

The three peptide-fluorescein-tagged RAFT agents **1**–**3** were synthesized using solid-phase methods (Scheme 1). Fmoc-Lys(Boc)–OH was sequentially coupled onto aminomethyl polystyrene resin, bearing a Rink-amide linker, using Oxyma and DIC as a coupling mixture until the desired peptide lengths were obtained. Fmoc-Lys(Dde)–OH was then coupled to allow orthogonal deprotection of its ε-amino group and subsequent coupling of the fluorophore onto its side chain. The Dde group was selectively removed by a mixture of hydroxylamine hydrochloride and imidazole,^31^ followed by the coupling of FAM. Finally, 2-(dodecylthiocarbonothioylthio)-2-methylpropionic acid (DDMAT), a carboxylic acid terminated trithiocarbonate RAFT agent, was conjugated onto the amino-terminus of the peptide. Cleavage off the resin and peptide deprotection using a mixture of TFA:H_2_O:TIS (90:5:5, *v/v/v*) proceeded cleanly without side-reaction between the RAFT agent and the reducing agent triisopropylsilane (TIS).

**Scheme 1:**
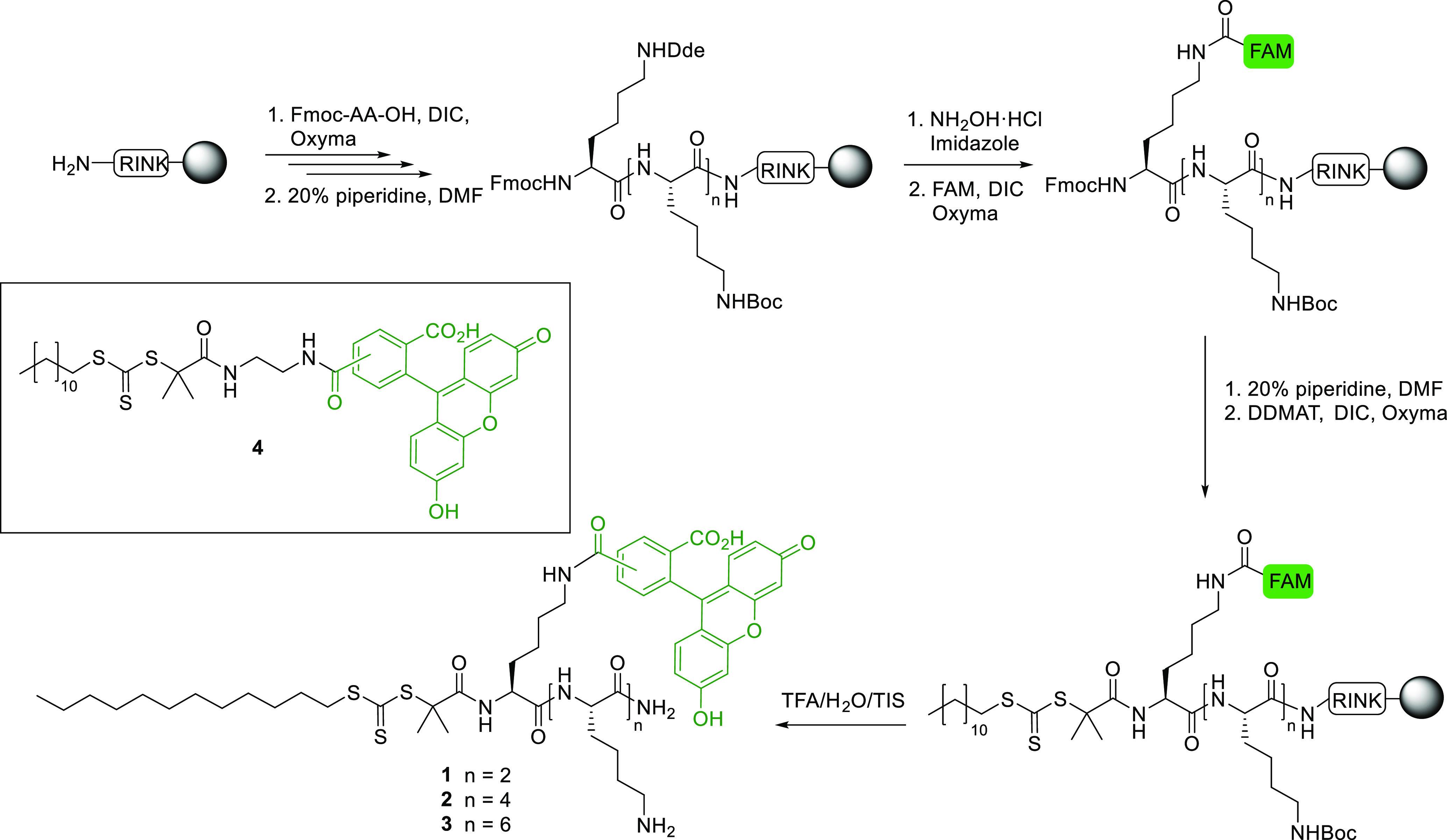
Solid-Phase Synthesis of the Fluorescein-Tagged Lysine RAFT Agents **1**–**3** Containing 3, 5, and 7 l-Lysine Residues and the Structure of the Control FAM-RAFT Agent **4**

The peptide-RAFT agents **1**–**3** were obtained with >99% purity after RP-HPLC purification and were fully characterized (see the Supporting Information). In addition, a fluorescein-tagged control RAFT agent **4** without any Lysine residues was synthesized in three steps using amide coupling between FAM and a DDMAT derivative prefunctionalized with a diaminoethane spacer (Scheme S1).

DMA was initially selected as the monomer to form the polymer chains due to its compatibility with the RAFT agent, the speed of polymerization, and the low PDIs previously reported with this monomer. Polydimethyl acrylamide (PDMA) also has high biocompatibility and stability toward hydrolysis,^35^ thus providing a reliable model polymer to validate the strategy. Here, polymers of 7.5 and 50 kDa were selected to span the molecular weight range and challenge the peptide delivery systems. Short polymers are less likely to be toxic and have higher initial uptake,^23^ while larger polymers (>40 kDa) have been shown to be have higher long-term accumulation in tumors.^36^

DMA was polymerized with RAFT agent **1**–**4** under similar conditions using AIBN as an initiator. Quenching at ∼95% monomer conversion yielded highly size-controlled and low PDI polymers: ∼7.5 kDa polymers **3Lys-PDMA-7.5k**, **5Lys-PDMA-7.5k**, and **7Lys-PDMA-7.5k** and ∼50 kDa polymers **3Lys-PDMA-50k**, **5Lys-PDMA-50k**, and **7Lys-PDMA-50k**, as well as the control polymers without the CCP (Table 1). The RAFT agents **1**–**4** polymerized at a rate similar to that of the unmodified DDMAT, indicating that the addition of the fluorophore or the peptide did not inhibit the rate of radical transfer. The polymer sizes were determined by ^1^H NMR (polymer backbone resonances integrated relative to the RAFT agent’s terminal CH_3_) and GPC (Figures S1 and S2). Determination of the final monomer conversion by ^1^H NMR allowed the calculation of the theoretical molecular weights as a percentage of the initial target molecular weight (i.e., 7.5 and 50 kDa) to which the mass of the RAFT agents was added. Polymers were also characterized by fluorescence spectroscopy with polymers of the same size showing similar fluorescence intensities at the same concentration (Figure S3). We evaluated the versatility of our peptide-RAFT agents by polymerizing different types of vinyl monomers (Scheme 2 and Table S1). In addition to the acrylamide monomer DMA, RAFT agent **1** successfully induced the controlled polymerization of hydrophilic 2-hydroxyethyl acrylate (2HEA) and aromatic styrene under identical conditions. ^1^H NMR and GPC characterization (Figures S4–S6) confirmed that **3Lys-P(2HEA)** was obtained with an *M*_w_ of ∼44 kDa reached in 4 h (Supporting Information).

**Table 1:**
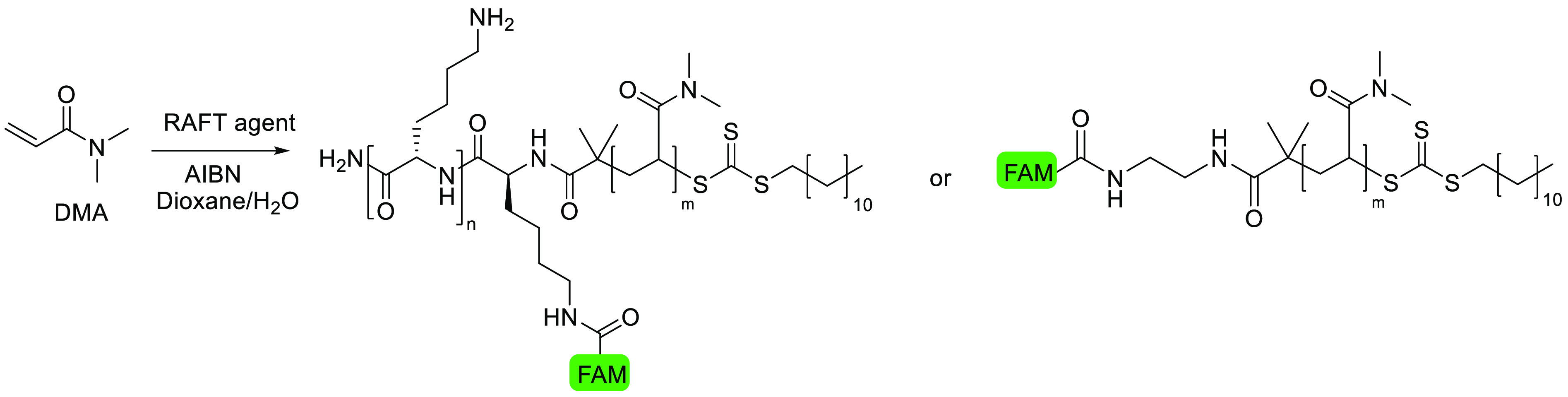
Synthesis and Characterization of the Fluorescently Tagged Lysine**–**PDMA Polymers Synthesized Using the RAFT Agents **1**–**4**

RAFT agent	Polymer	*n*	Conv. [%]^a^	Theor. *M*_w_ [kDa]^b^	*M*_w_ [kDa] (^1^H NMR)	*M*_w_ [kDa]^c^ (GPC)	PDI
**1**	**3Lys-PDMA-7.5k**	2	97	8.3	8.2	7.9	1.15
**2**	**5Lys-PDMA-7.5k**	4	98	8.6	8.5	8.0	1.41
**3**	**7Lys-PDMA-7.5k**	6	97	8.8	8.8	10	1.30
**4**	**0Lys-PDMA-7.5k**	–	96	7.8	7.7	6.2	1.04
**1**	**3Lys-PDMA-50k**	2	97	49	53	49	1.35
**2**	**5Lys-PDMA-50k**	4	98	50	52	47	1.27
**3**	**7Lys-PDMA-50k**	6	98	50	52	52	1.26
**4**	**0Lys-PDMA-50k**	–	96	48	49	46	1.30

a Monomer conversion determined by ^1^H NMR.

b Based on monomer conversion and the mass of the RAFT agent.

c Determined by GPC using DMF with 0.1% LiBr as eluent and PMMA as reference standards. The polymer size discrepancies in the GPC analyses can be attributed to the charge differences between the polymers, intermolecular interactions,^32−34^ and discrepancies arising from the reference used.

**Scheme 2:**
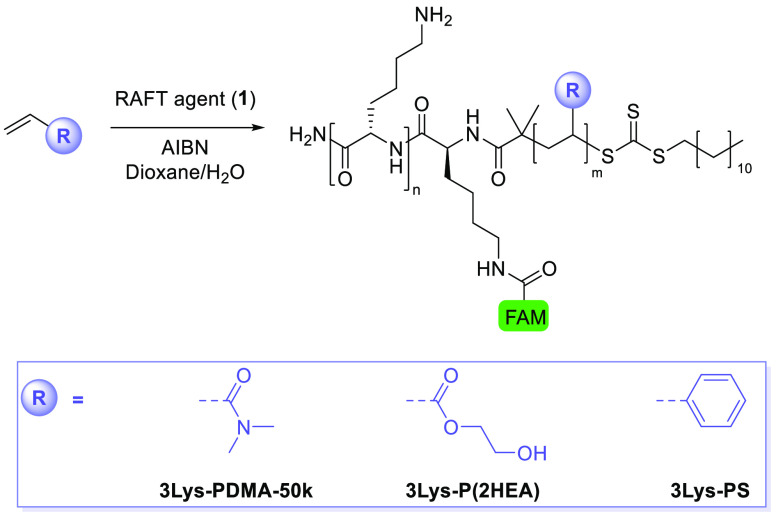
Synthesis of the Fluorescently-Tagged, Lysine–Polymer Conjugates Using RAFT Agent **1** and Various Vinyl Monomers

As expected, styrene polymerized at a slower rate under these conditions (52% conversion in 48 h, as determined by ^1^H NMR), giving the polymer **3Lys-PS** with a molecular weight of ∼30 kDa.

To evaluate cellular uptake efficiency, HeLa and MCF-7 cells were incubated with the 7.5 and 50 kDa polymers (50 μg/mL) overnight and analyzed by flow cytometry (Figures 1a,b and S7). For the low molecular weight peptide–PDMAs, the poly-Lysine chain length had a large impact on polymer uptake into HeLa cells (Figures 1a and S7). The longer peptides resulted in higher polymer uptake with 1.7, 3.4, and 6.0-fold increases in uptake for **3Lys-PDMA-7.5k**, **5Lys-PDMA-7.5k**, and **7Lys-PDMA-7.5k**, respectively, compared to the control polymer (**0Lys-PDMA-7.5k**). The 50 kDa polymers **3Lys-PDMA-50k**, **5Lys-PDMA-50k**, and **7Lys-PDMA-50k** also showed an increased uptake with poly-Lysine chain length (1.1, 1.5, and 2.1-fold increase in fluorescence, respectively). This was slightly less significant than with the smaller polymers, likely due to the higher initial uptake of the shorter polymers. An identical uptake pattern was observed on MCF-7 cells (Figure S8), with similar fold increases in uptake with increased poly-Lysine length and between short and long PDMAs.

**Figure 1:**
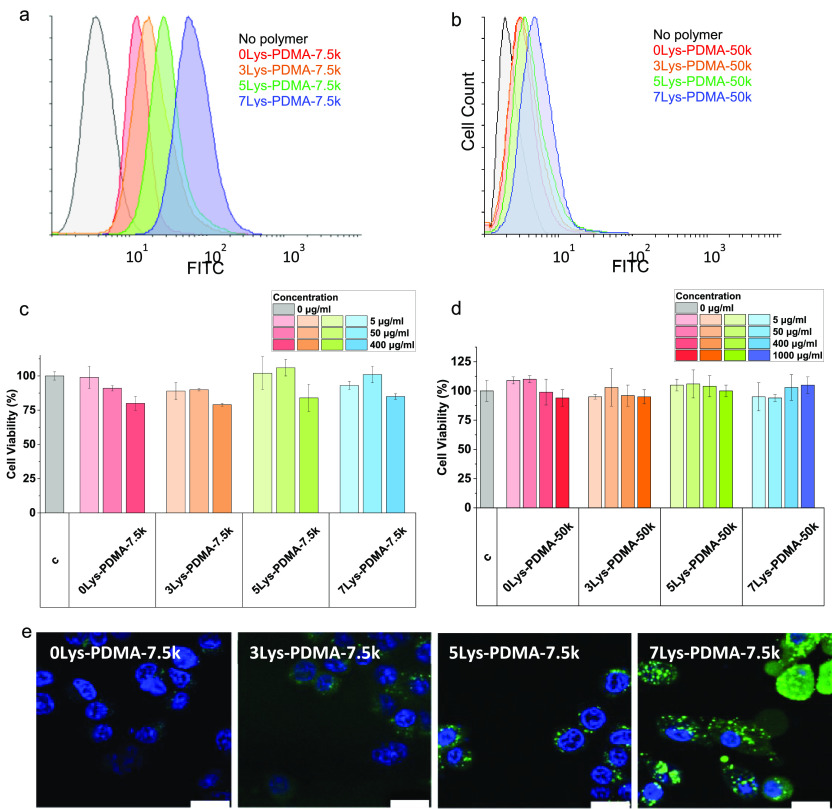
Biocompatibility, cellular uptake, and localization studies of the fluorescently tagged peptide–polymer conjugates in HeLa cells. (a, b) Flow cytometry histograms showing an increase in the cellular uptake of both the 7.5 and 50 kDa polymers with Lysine number on the fluorescein channel (λ_Ex_ = 488 nm, λ_Em_ = 530/30 nm) compared to the corresponding control polymer. (c, d) Cell viability (MTT assay) of HeLa cells after incubation with the 7.5 kDa (5–400 μg/mL, 24 h) and 50 kDa polymers (5–1000 μg/mL, 24 h). c = control. Values are mean ± SD, *n* = 3, *p* > 0.05. (e) Confocal images of the **0Lys-PDMA-7.5k** control, **3Lys-PDMA-7.5k**, **5Lys-PDMA-7.5k**, and **7Lys-PDMA-7.5k** polymers (green, λ_Ex/Em_ = 492/517 nm) in HeLa cells costained with Hoechst 33342 (nuclear stain) (blue, λ_Ex/Em_ = 392/440 nm). All images were acquired using the same gain and exposure times. Scale bar: 20 μm.

Interestingly, although higher increases in uptake have been reported with Penetratin and TAT-polymer conjugates,^37^ this fold increase in cellular uptake is of similar magnitudes to values reported using more complex RAFT-CPPs such as Transportan 10,^21^ which highlights the potential of this straightforward RAFT design. Importantly, the short polymer–poly-Lysine conjugates also showed good biocompatibility in MTT cytotoxicity assays (5–400 μg/mL, Figures 1c and S8e). The longer polymers showed no toxicity on HeLa and MCF-7 cell lines, even at very high concentrations (up to 1000 μg/mL; Figures 1d and S8f). We hypothesized that increasing the concentration of the short polymers could potentially result in a lower cell viability compared to the longer polymers because of a higher positive charge density within the cells; however, both cell lines retained high cell viability with charge density, even at high polymer concentration.

Cell uptake of the PDMA–peptide conjugates was also evaluated by using confocal microscopy (Figures 1e and S9). HeLa cells were incubated with the ∼7.5 kDa polymers for 24 h and costained with Hoechst 33342. A large increase in fluorescence intensity was seen for the **3**, **5**, and **7 Lys-PDMA-7.5k** polymers compared to the control polymer, with the fluorescence intensity proportional to the number of Lysine residues (Figure S10). Finally, the cellular localization of the polymer–peptides conjugates was explored due to reports that poly-Lysine moieties enter cells via nonspecific adsorptive endocytosis.^38^ Endosomal localization was confirmed by costaining cells with the endosomal stain CellLight Early Endosomes-RFP and indicated that the polymer–peptides were up taken by this pathway (Figure S11). The possibility of the constructs forming nanoparticles and having a critical micelle concentration was explored by performing a Nile Red fluorescence assay (Figure S12).^39^ For all of the peptide–PDMA samples and corresponding controls, no increase in Nile Red fluorescence was observed across a range of polymer concentrations, confirming that no encapsulation/nanoparticle formation was occurring. This confirms that the peptide–PDMA polymers do not self-assemble into nanoparticles even at concentrations higher than those used in our cell uptake experiments and that the samples only contain the free polymer chains in solution.

In summary, trackable polymer conjugates were prepared by a controlled polymerization reaction using fluorescently tagged poly-Lysine-based RAFT agents. These new CPP-RAFT agents, prepared entirely by solid-phase synthesis, efficiently promoted the polymerization of long and short acrylamides as well as acrylate and styrene monomers with excellent size and PDI control. A 6-fold increase in polymer cell uptake was achieved as a result of adding Lysine residues as an end group to the polymer chains, which is higher than uptake values reported for other, more complex CPP moieties, and these Lysine-based RAFT agents can therefore be used as a strategy to deliver polymer vehicles inside cells for therapeutic and diagnostic applications. Importantly, these CPP-RAFT agents were thermally and chemically resistant, and the polymerization was performed efficiently without the need to keep protecting groups on the amino acids. These RAFT agent designs are accessible, versatile, and convenient tools for the synthesis of new polymer–peptide conjugates as they enable simultaneous fluorescence labeling in a highly controlled manner (i.e., a single fluorophore per polymer chain) and without any postfunctionalization steps. The resulting fluorescently tagged polymer–peptide conjugates can help to tackle the challenges of drug bioavailability and to develop enhanced therapies. Drug–polymer conjugates, such as PEGAdagen and Eudragit, have shown that polymers can improve drug circulation time and bioavailability,^5^ or introduce controlled release properties.^6^ Combining drug conjugation with our peptide–polymer conjugates could lead to constructs with optimal cell-delivery abilities, further improving pharmacological properties, while monomers containing trigger-sensitive prodrugs could also be copolymerized. In an alternate strategy, the synthesis of amphiphilic diblock polymers from our CPP-RAFT agents would also enable the preparation of peptide-coated nanoparticles, allowing efficient cargo delivery. Our RAFT agents could facilitate cellular delivery of polymeric drug conjugates, determination of their cellular localization, and tuning of physiological cellular parameters such as viscosity and cell proliferation.

## Supporting Information Available

Additional synthetic scheme, materials, methods, and experimental and characterization details, including ^1^H NMR, HPLC, and HR-MS data of new compounds; GPC data for polymers; and additional flow cytometry and confocal microscopy data (PDF)
