# Supplementary material for: Fluorophore-Tagged Poly-Lysine RAFT Agents: Controlled Synthesis of Trackable Cell-Penetrating Peptide–Polymers
Source: ACS Macro Lett. 2023 Sep 11;12(10):1280–5. doi: 10.1021/acsmacrolett.3c00460 (PMC10586461; doi:10.1021/acsmacrolett.3c00460)
Supplement: Supplementary file 1 — mz3c00460_si_001.pdf [file mz3c00460_si_001.pdf]

Fluorophore-tagged poly-Lysine RAFT agents: Controlled synthesis of trackable cell-penetrating polymers

Paige A. Shaw,[1] Maxime Klausen,[1] Annamaria Lilienkampf,[1] and Mark Bradley[1,2,*]

[1] EaStCHEM School of Chemistry, University of Edinburgh, David Brewster Road, EH9 3FJ, Edinburgh, UK.

[2] Precision Healthcare University Research Institute, Queen Mary University of London, 65-67 New Road, E1 1HH, London, UK. E-mail: m.bradley@qmul.ac.uk.

Table of Content

1. Supporting figures and schemes S2
2. Experimental section S11
   2.1. Materials and Methods S11
   2.2. Solid-phase synthesis of fluorescein tagged Peptide-RAFT agents 1–3 ....S11
   2.3. Synthesis of Control RAFT agent 4 ....S15
   2.4. Polymerisation reactions S16
   2.5. Polymer characterisation S17
   2.6. Biological Assays S18
3. NMR, HRMS (MALDI), and HPLC characterisations S20
4. Supplementary references S26

1. Supporting figures and schemes

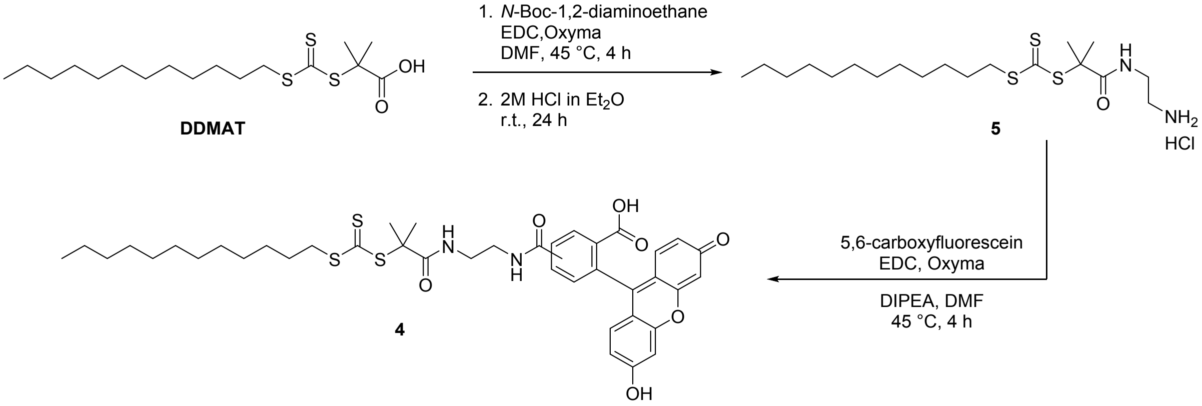

Scheme S1. Synthesis of the control RAFT-agent **4** without Lysines.

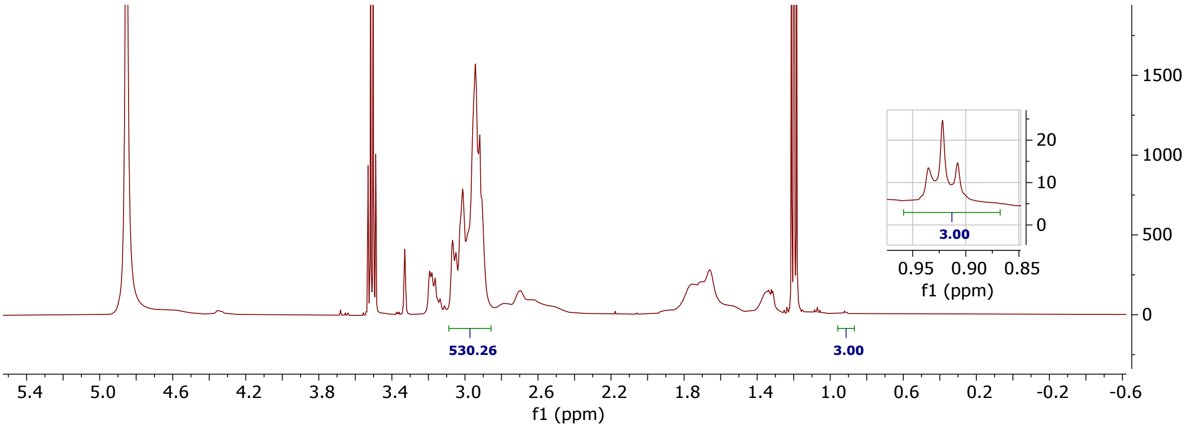

Figure S1. Determination of the size of **3Lys-PDMA-7.5k** by ^{1}H NMR spectroscopy. The resonance at 2.85 – 3.10 ppm (PDMA chain) were integrated with respect to the triplet at 0.92 ppm (terminal CH_3 on the RAFT agent). The same method was applied for the size determination of all the polymers.

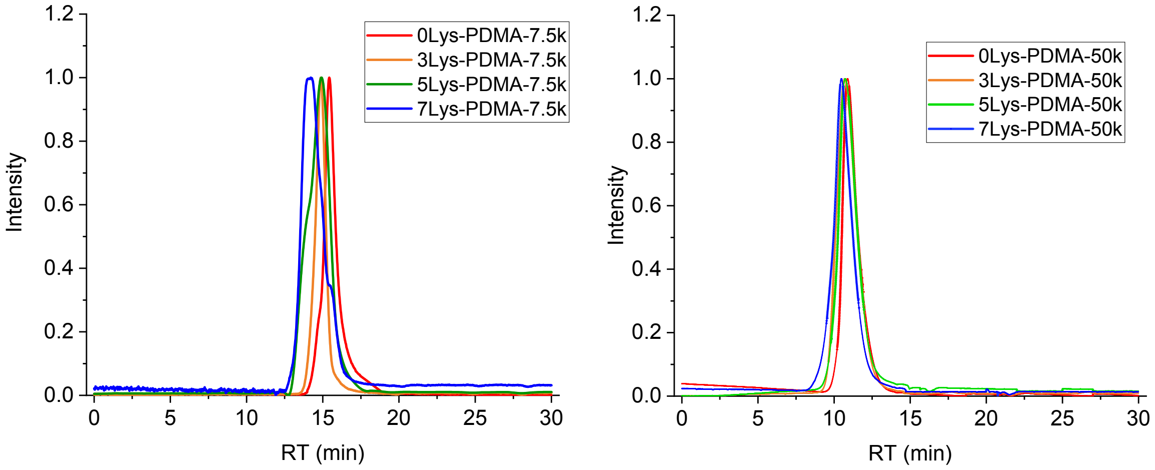

Figure S2. GPC traces of the 7.5 kDa and 50 kDa Lysine–PDMA polymers.

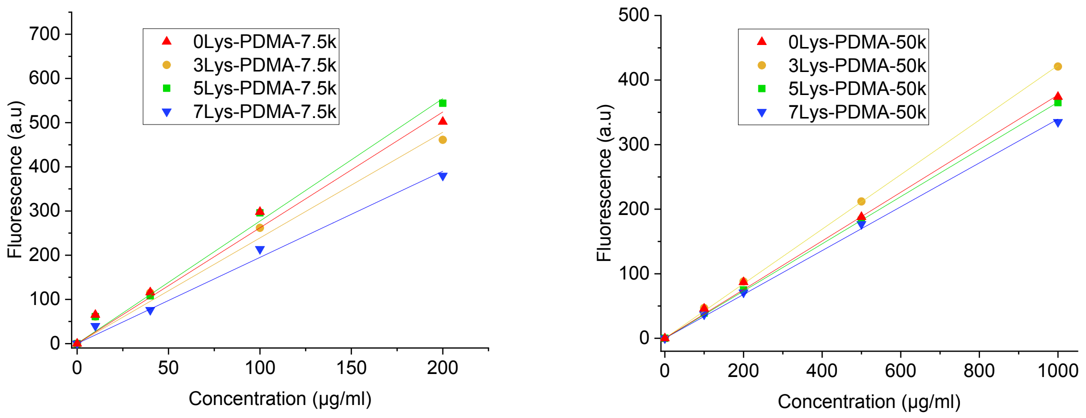

Figure S3. Concentration dependent fluorescence ($\lambda_{Ex/Em}$ = 480/520 nm) of the 7.5 and 50 kDa polymers in PBS (prepared by serial dilutions of 2 mg/mL stock solutions).

Table S1. Synthesis and characterization of fluorescently-tagged, Lysine–polymer conjugates synthesized using RAFT agent **1** and 2HEA and styrene as monomers.

Monomer	Polymer	Conv. [%][a]	Theor. Mw [kDa][b]	Mw [kDa] (^{1}H NMR)	Mw [kDa][c] (GPC)	PDI
2HEA	**3Lys-P(2HEA)**	75	44	44	42	1.47
Styrene	**3Lys-PS**	52	28	28	31	1.59

[a] Monomer conversion determined by ^{1}H NMR. [b] Based on monomer conversion and the mass of the RAFT agent. [c] Determined by GPC using DMF with 0.1% LiBr as eluent and PMMA as reference standards.

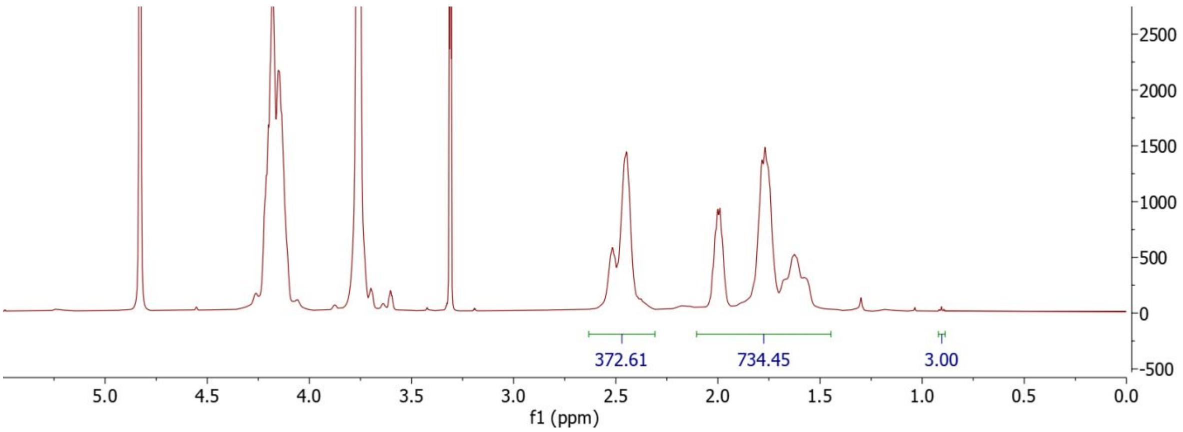

Figure S4: Determination of the size of **3Lys-P(2HEA)-50k** by ^{1}H NMR spectroscopy in deuterated methanol. The resonance at 2.67 – 2.27 ppm and 2.05 – 1.55 ppm (2HEA backbone) were integrated with respect to the triplet at 0.91 ppm (terminal CH_3 on the RAFT agent).

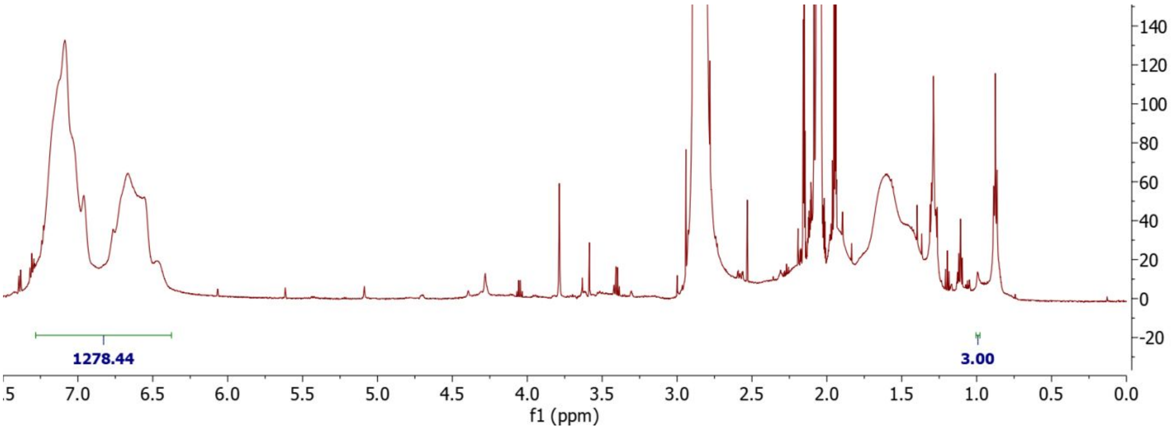

Figure S5: Determination of the size of **3Lys-PS-50k** by ^{1}H NMR spectroscopy in deuterated acetone. The resonance at 7.37 – 6.31 ppm (Styrene rings) were integrated with respect to the triplet at 0.98 ppm (terminal CH_3 on the RAFT agent).

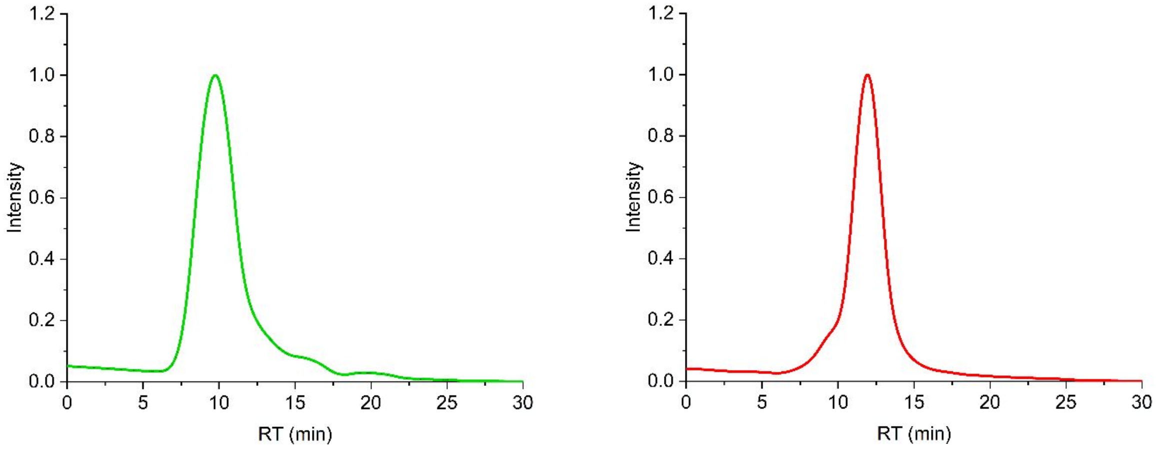

Figure S6. GPC traces of the 50 kDa **3Lys-P(2HEA)-50k** (left) and **3Lys-PS-50k** (right).

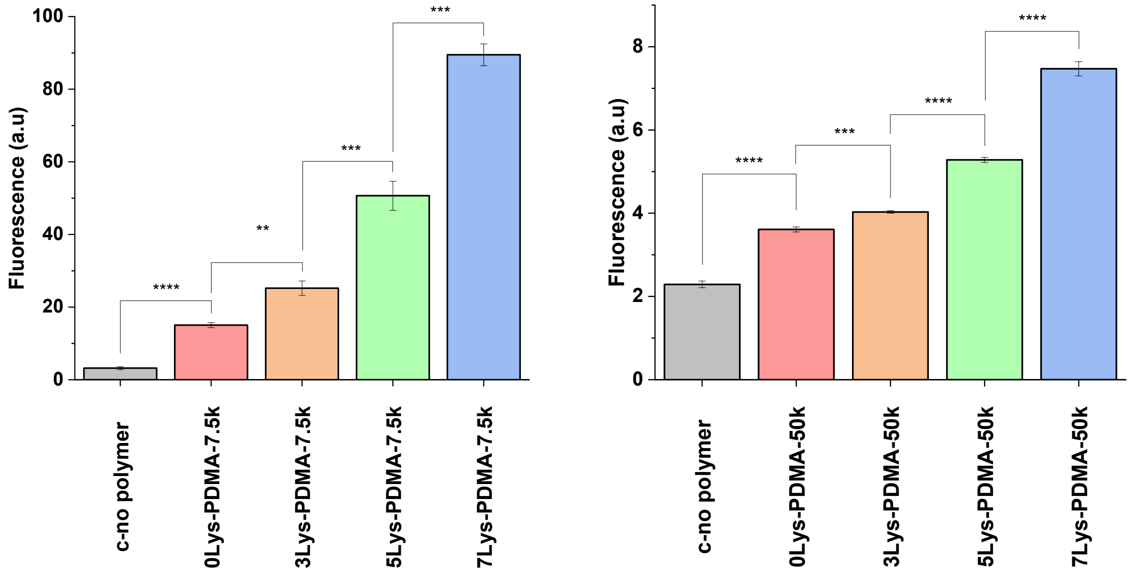

Figure S7. The cellular uptake (HeLa cells) of the 7.5 kDa and 50 kDa polymers as measured by flow cytometry. The cells were incubated with the polymers at 50 µg/mL for 24 h. Values are mean ± SD, n = 3, ** = $p \leq 0.01$, *** = $p \leq 0.001$, **** = $p \leq 0.0001$.

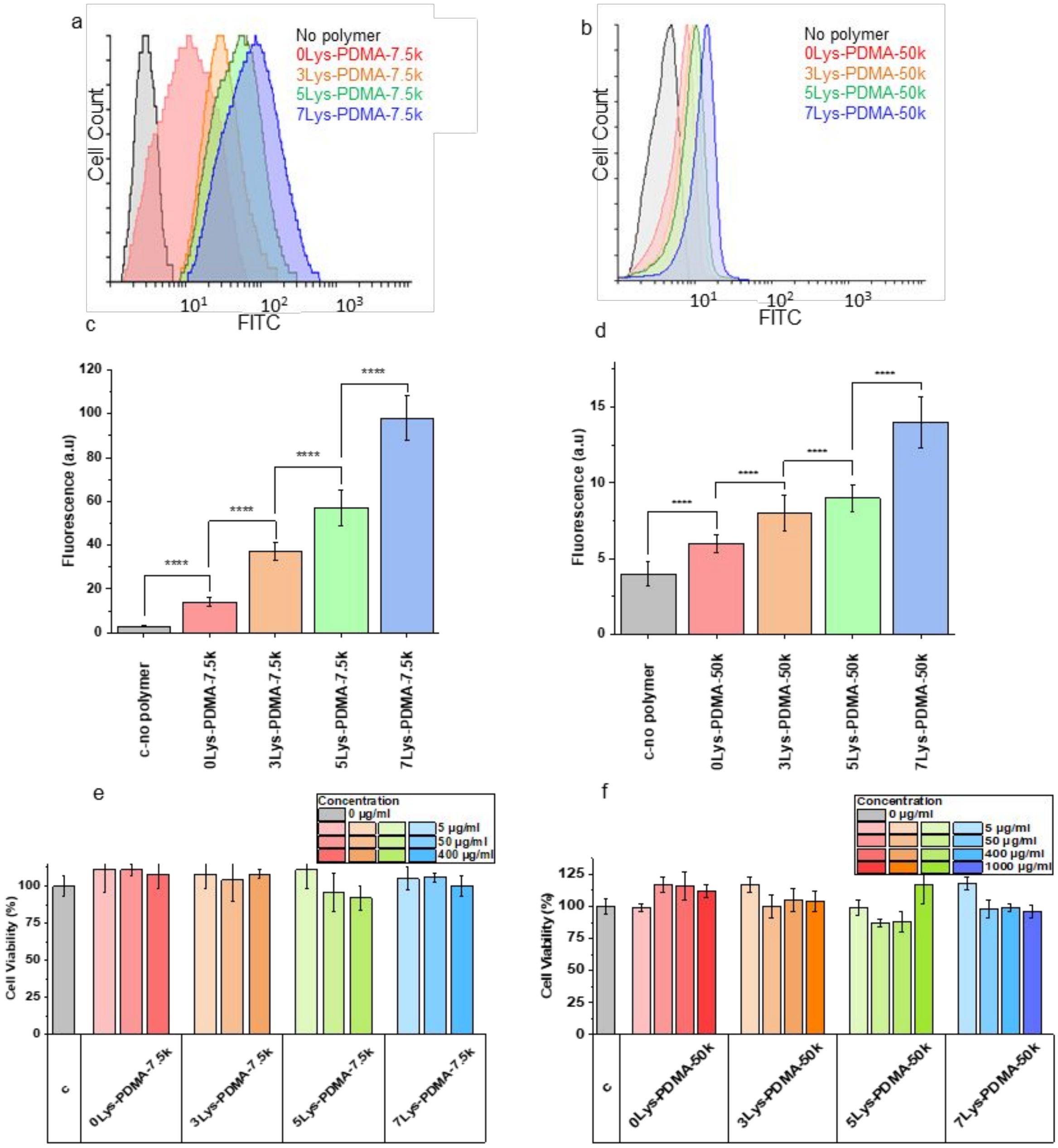

Figure S8: Cellular uptake and viability study of the peptide polymers in MCF7 cells. a, b) Flow cytometry histograms showing an increase in the cellular uptake of both the 7.5 kDa and 50 kDa polymers (50 µg/ml) with Lysine number on the fluorescein channel (λ_{Ex} = 488 nm, λ_{Em} = 530/30 nm) compared to the corresponding control polymer. c, d) Cellular uptake of the 7.5 kDa and 50 kDa polymers as measured by flow cytometry. Values are mean ± SD, n = 3, **** = p ≤ 0.0001. e, f) Cell viability (MTT assay) of MCF7 cells after incubation with the 7.5 kDa (5–400 µg/mL, 24 h) and 50 kDa polymers (5–1000 µg/mL, 24 h).

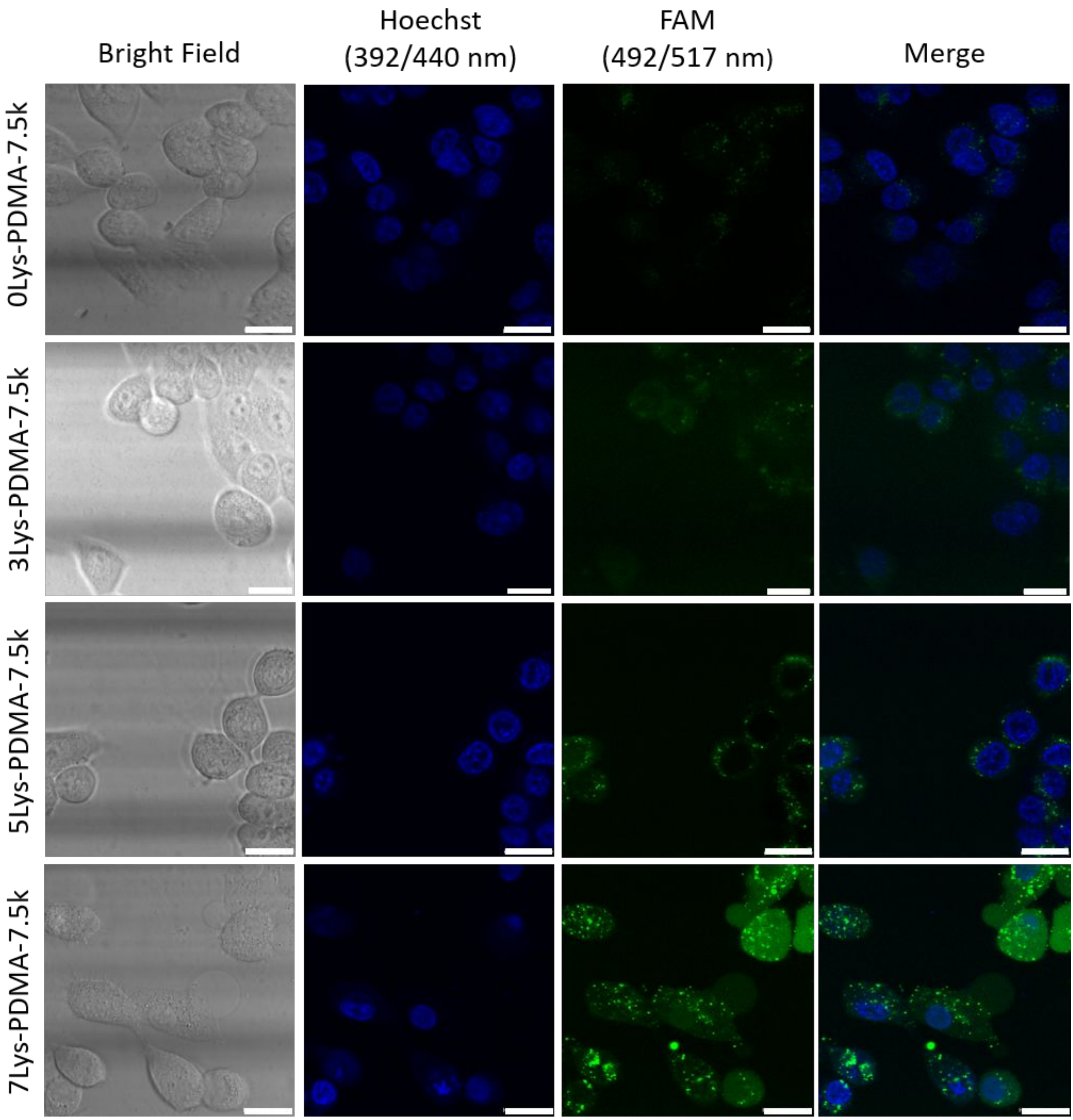

Figure S9 Confocal microscopy images of HeLa cells following incubation with 200 µg/mL (24 h) of the 7.5 kDa polymers (FAM channel, $\lambda_{Ex/Em}$ = 492/517 nm). The cells were co-stained with Hoechst 33342 nuclear stain ($\lambda_{Ex/Em}$ = 392/440 nm). Scale bar = 20 µm.

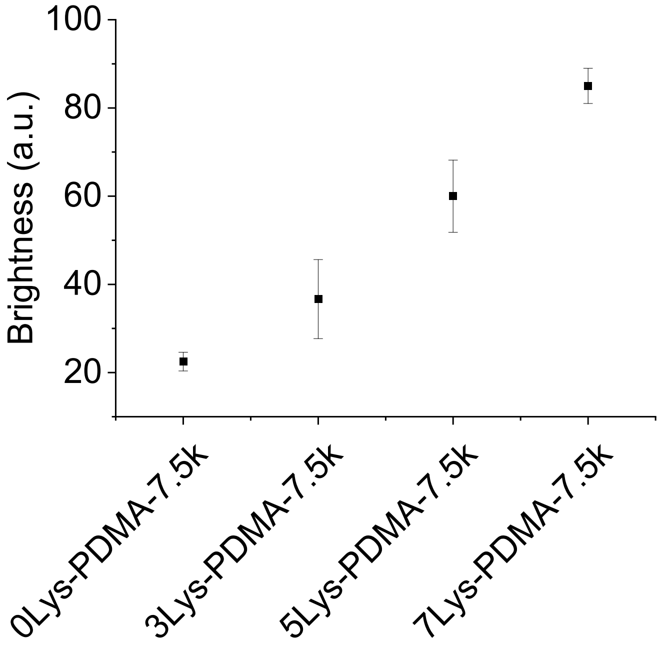

Figure S10. Comparison of the brightness of HeLa cells following incubation with 200 µg/mL (24 h) of the 7.5 kDa polymers (FAM channel, $\lambda_{Ex/Em}$ = 492/517 nm). Brightness values were determined by analyzing the confocal microscopy images using ImageJ.

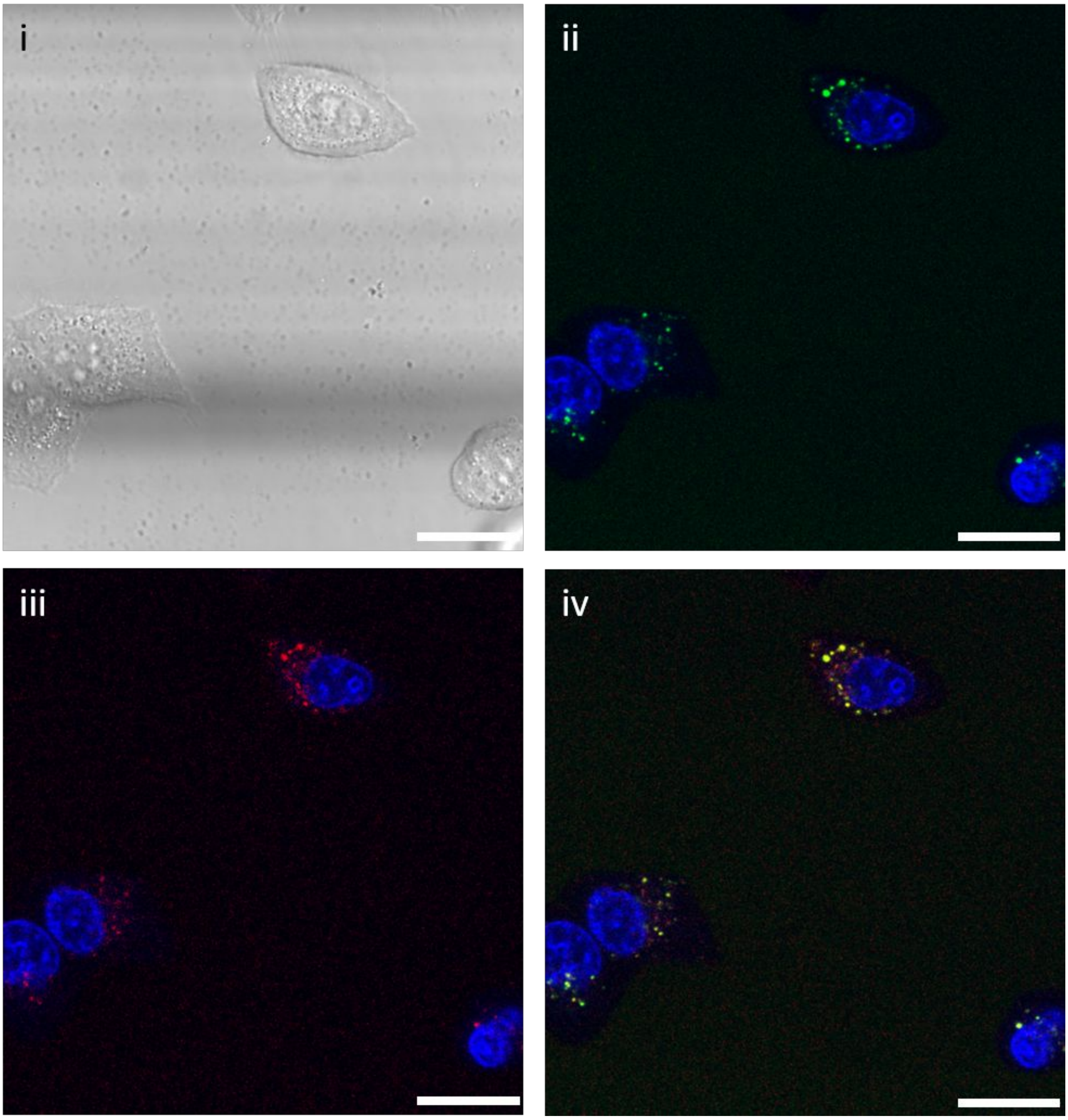

Figure S11. Confocal microscopy images of HeLa cells showing colocalization of the polymer (green, $\lambda_{Ex/Em}$ = 492/517 nm) with endosomes. HeLa cells were incubated with **5Lys-PDMA-7.5k** (200 µg/mL, 24 h), co-stained with Hoechst 33342 nuclear stain (blue, $\lambda_{Ex/Em}$ = 392/440 nm) and with CellLight® early Endosomes-RFP, BacMam 2.0 (ThermoFisher) for staining of early endosomes (red, $\lambda_{Ex/Em}$ = 555/584 nm). (i) Bright field. (ii) Merged blue and green channels. (iii) Merged blue and red channels. (iv) Merged blue, green and red channels. Scale bar = 20 µm. Pearson's Coefficient r = 0.431. Mander's coefficient (fraction of (ii) overlapping (iii)) = 0. 994.

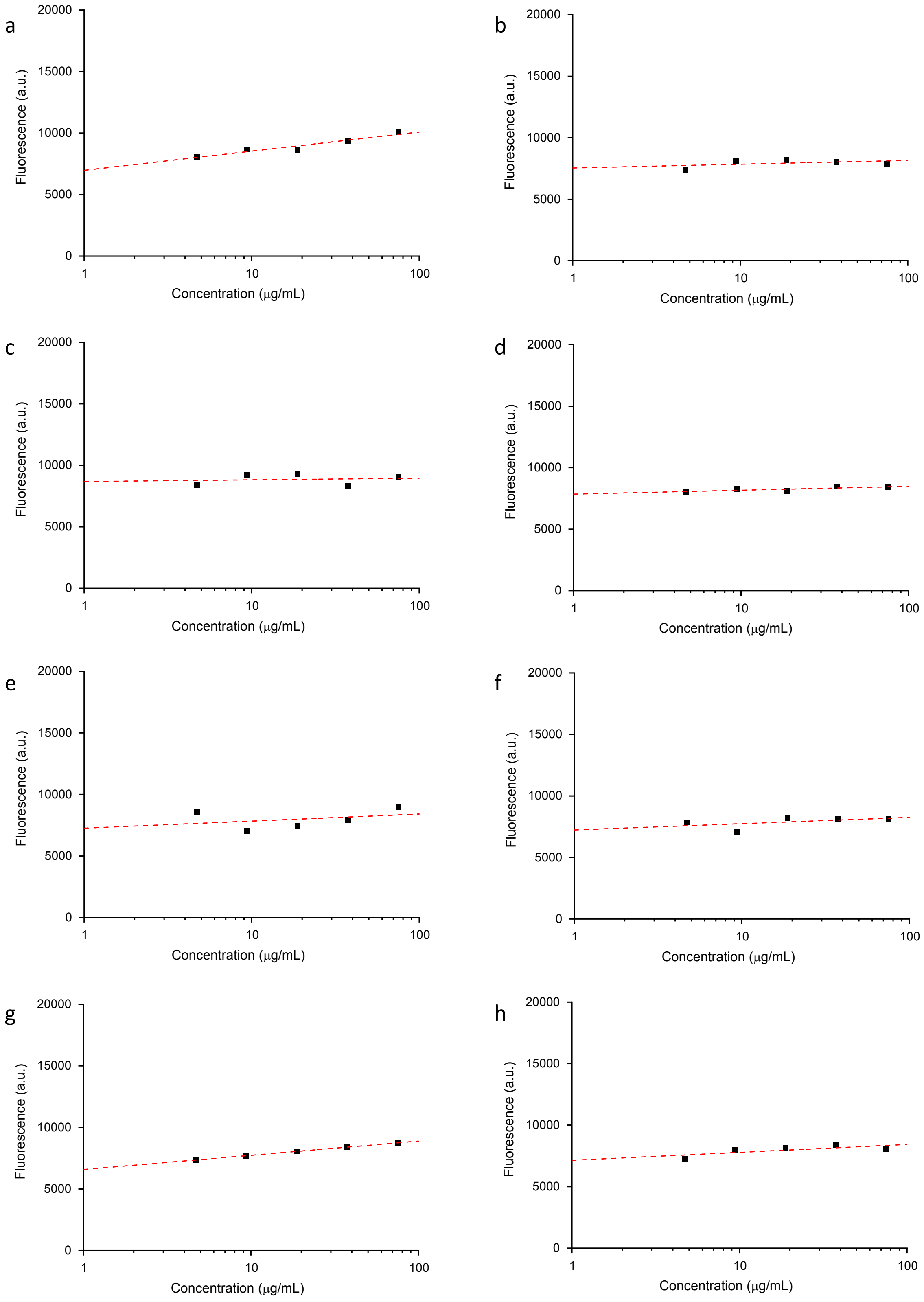

Figure S12. Fluorescence dependence of Nile Red ($\lambda_{Ex/Em}$ = 610/655 nm) in the presence of increasing concentrations of a) **0Lys-PDMA-7.5k**, b) **0Lys-PDMA-50k**, c) **3Lys-PDMA-7.5k**, d) **3Lys-PDMA-50k**, e) **5Lys-PDMA-7.5k**, f) **5Lys-PDMA-50k**, g) **7Lys-PDMA-7.5k** and h) **7Lys-PDMA-50k** (4.7 – 75 µg/mL) in H_2O.

2. Experimental section

2.1. Materials and Methods

Fmoc-protected amino acids and the aminomethyl polystyrene resin were purchased from GL Biochem (Shanghai) Ltd and NovaBiochem, cell culture reagents were from Sigma Aldrich, Corning, Life Technology, and Thermo Fisher, and all other chemicals from Sigma Aldrich and Acros. AIBN was recrystallized from MeOH and monomers (DMA, 2HEA and styrene) were filtered through a plug of basic alumina to remove inhibitors prior to use.

^{1}H and ^{13}C NMR spectra were recorded on a Bruker AVA-500 (at 500 and 125 MHz, respectively) at 298 K in the deuterated solvents indicated. Shifts (δ) are given in parts per million (ppm) with respect to solvent residual peak, and coupling constant (*J*) are given in Hertz. Analytical HPLC was performed on an Agilent Technologies 1100 modular HPLC system coupled to a multiple wavelength and ELSD detector and equipped with a Phenomenex Kinetex® 5µm XB-C18 100 Å column (50 × 4.6 mm). Samples were eluted with a gradient of H_2O/MeCN (buffered with 0.1% formic acid) from 95/5 to 5/95, over 6 min, then holding at 95% for 3 min, followed by elution at 5% MeCN for 1 min. Preparative RP–HPLC was performed on an Agilent 1100 system equipped with a Kinetex XB-C18 column (150 × 21.2 mm, 5 µm) with a flow rate of 10 mL/min and eluting with a gradient of H_2O/MeOH (buffered with 0.1% TFA) from 50/50 to 10/90 over 30 min. GPC was performed on an Agilent 1100 GPC equipped with PLgel MIXED-C columns (Range of MW $2 \times 10^2 – 2 \times 10^6$ g/mol, 5 µm) and an RI detector, eluting with DMF containing 0.1 % w/v LiBr at 60 °C and a flow rate of 1 mL/min. Electrospray ionization mass spectrometry (ESI–MS) analyses were carried out on an Agilent Technologies LC/MSD Series 1100 quadrupole mass spectrometer (QMS) in ESI mode. MALDI-ToF mass spectra were obtained with a Bruker UltraflexExtreme MALDI TOF/TOF instrument and analyzed with the Bruker Daltonics flexAnalysis software with a matrix solution of sinapic acid (10 mg/mL) in H_2O/CH_3CN/TFA (50/50/0.1). Fluorescence and absorbance measurements were performed on a BioTek HT Synergy multi-mode reader. Flow cytometry analysis was carried out on a Becton Dickinson (BD) FACSAria™ and analyzed using FlowJo.

2.2. Solid-phase synthesis of fluorescein tagged Peptide-RAFT agents 1–3

Each peptide was synthesized on a 1 g scale of an aminomethyl polystyrene resin with a loading of 0.642 mmol/g (100-200 mesh). All the coupling reactions were monitored using a Kaiser test.

Rink-amide linker attachment to Polystyrene Resin. Fmoc-Rink-amide linker (1.06 g, 1.9 mmol, 3 equiv.) and Oxyma (0.27 g, 1.9 mmol, 3 equiv.) were dissolved in DMF (10 mL) and the mixture was stirred for 10 min until fully dissolved. DIC (243 µL, 1.9 mmol, 3 equiv.) was added to the solution and the mixture was stirred for a further 2 min. This mixture was added to the resin (1.0 g, 0.642 mmol/g, 1 equiv., pre-swollen in DCM) and shaken for 1 h. The resin was washed with DMF (3 × 10 mL), DCM (3 × 10 mL), MeOH (3 × 10 mL). Upon successful coupling, the Fmoc group was removed using a solution of piperidine in DMF (20%, *v/v*, 20 mL) for 2 × 20 minutes. The solution was drained and the resin was washed with DMF (3 × 20mL), DCM (3 × 20mL), and MeOH (3 × 20mL).

Fmoc-Lys(Boc)-OH couplings. A solution of Fmoc-Lys(Boc)-OH (0.867 g, 1.9 mmol, 3 equiv. and Oxyma (0.27 g, 1.9 mmol, 3 equiv.) were dissolved in DMF (10 mL) and the mixture was stirred for 10 min until fully dissolved. DIC (243 µL, 1.9 mmol, 3 equiv.) was added to the solution and the mixture was stirred for a further 2 min. The mixture was added to the aforementioned resin (1.0 equiv., pre-swollen in DCM) and shaken for 1 h. The solution was drained and the resin was washed with DMF (3 × 20mL), DCM (3 × 20mL), MeOH (3 × 20mL). Upon successful coupling, Fmoc groups were removed using a solution of piperidine in DMF (20%, *v/v*, 20 mL) for 2 × 20 minutes. The solution was drained and the resin was washed with DMF (3 × 20mL), DCM (3 × 20mL), MeOH (3 × 20mL). The conjugation of additional Fmoc-Lys(Boc)-OH residues (2, 4, and 6) gave peptides **1**, **2**, and **3**, respectively.

Fmoc-Lys(Dde)-OH couplings and Dde deprotection. Fmoc-Lys(Dde)-OH[1] (0.743 g, 1.9 mmol, 3 equiv.) and Oxyma (0.27 g, 1.9 mmol, 3 equiv.) were dissolved in DMF (10 mL) and the mixture was stirred for 10 min until fully dissolved. DIC (243 µL, 1.9 mmol, 3 equiv.) was then added to the solution and the mixture was stirred for a further 2 min. The mixture was added to the aforementioned resin (1.0 equiv., pre-swollen in DCM) and stirred for 1 h. The solution was drained and the resin was washed with DMF (3 × 20mL), DCM (3 × 20mL), MeOH (3 × 20mL). The Dde group was then removed from the side chain by using a solution of imidazole (4.05 mmol, 0.27 M) and hydroxylamine hydrochloride (5.4 mmol, 0.36 M) in NMP (15 mL). This mixture was diluted by adding DCM (3 mL) and added to the resin, and shaken for 1.5 h. The deprotection was repeated 3 times. The solution was drained and the resin was washed with DMF (3 × 20mL), DCM (3 × 20mL), MeOH (3 × 20mL).

5,6-Carboxyfluorescein coupling. 5,6-Carboxyfluorescein (0.724 g, 1.9 mmol, 3 equiv.) and Oxyma (0.27 g, 1.9 mmol, 3 equiv.) in DMF (10 mL) were stirred for 10 min until fully dissolved. DIC (243 µL, 1.9 mmol, 3 eq) was added to the solution and the mixture was stirred for a further 2 min. The mixture was added to the aforementioned resin (1.0 equiv., pre-swollen in DCM) and shaken for 4 h. The solution was drained and the resin was washed with DMF (3 × 20mL), DCM (3 × 20mL), MeOH (3 × 20mL). Upon successful coupling, the Fmoc group was removed from the N-terminus using a solution of piperidine in DMF (20%, *v/v*, 20 mL) for 2 × 20 minutes. The solution was drained and the resin was washed with DMF (3 × 20mL), DCM (3 × 20mL), MeOH (3 × 20mL).

RAFT agent coupling. 2-(Dodecylthiocarbonothioylthio)-2-methylpropionic acid (0.365 g, 1.9 mmol, 3 equiv.) and Oxyma (0.27 g, 1.9 mmol, 3 equiv.) were dissolved in DMF (10 mL) and the mixture was stirred for 10 min until fully dissolved. DIC (243 µL, 1.9 mmol, 3 equiv.) was added to the solution and the mixture was stirred for a further 2 min. The mixture was added to the aforementioned resin (1.0 equiv., pre-swollen in DCM) and shaken for 1 h. The solution was drained, and the resin was washed with DMF (3 × 20mL), DCM (3 × 20mL), MeOH (3 × 20mL).

Cleavage off the resin, deprotection and purification. A solution of TFA/TIS/H_2O (15 mL) in a ratio of 90:5:5 (*v/v/v*) was added to the resin (pre-swollen in DCM) and the mixture was shaken for 4 h. The solution was drained and the product precipitated into ice-cold Et_2O and collected by centrifugation to give the crude peptide-RAFT agents. The peptide-RAFT agents were then dissolved (~30 mg/mL) in

H_2O with 0.1% TFA and purified by reverse-phase preparative-HPLC (see materials and methods). Fractions were collected and freeze-dried to give the final peptide–RAFT agents as yellow solids.

3-Lys-FAM-RAFT (1)

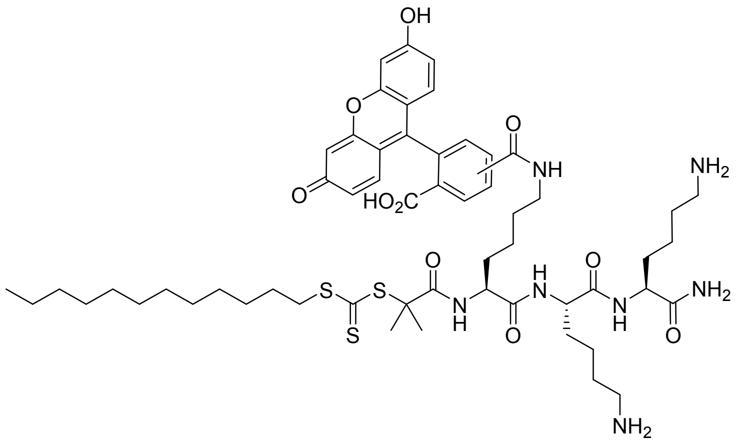

Yield 11% (76 mg, yellow powder). ^{1}H NMR (500 MHz, Methanol-d_4) δ 8.47 – 8.43 (m, 0.5H, *isomer 5*), 8.22 (dd, *J* = 8.0, 1.6 Hz, 0.5H, *isomer 5*), 8.14 (dd, *J* = 8.0, 1.4 Hz, 0.5H, *isomer 6*), 8.11 – 8.06 (m, 0.5H, *isomer 6*), 7.68 – 7.64 (m, 0.5H, *isomer 6*), 7.31 (d, *J* = 7.8 Hz, 0.5H, *isomer 5*), 6.72 – 6.65 (m, 2H), 6.65 – 6.48 (m, 4H), 4.40 – 4.27 (m, 4H), 4.19 (dd, *J* = 8.4, 5.9 Hz, 0.5H, *isomer 5*), 4.10 (dd, *J* = 8.5, 5.8 Hz, 0.5H, , *isomer 6*), 3.00 – 2.87 (m, 6H), 1.95 – 1.40 (m, 26H), 1.40 – 1.20 (m, 20H), 0.94 – 0.84 (m, 3H); ^{13}C NMR (126 MHz, Methanol-d_4) δ 222.9, 176.5, 175.3, 175.2, 174.6, 174.56, 173.76, 173.73, 170.53, 170.48, 168.4, 168.3, 161.7, 154.2, 154.1, 142.3, 137.9, 135.5, 130.4, 130.4, 130.4, 130.2, 130.2, 128.7, 126.3, 125.8, 125.2, 124.5, 114.0, 113.9, 111.01, 110.96, 103.7, 57.8, 57.6, 56.2, 56.1, 54.44, 54.38, 53.98, 53.96, 40.58, 40.56, 40.51, 40.49, 40.4, 37.8, 37.7, 33.05, 33.04, 32.7, 32.6, 32.20, 32.18, 32.15, 32.0, 31.8, 30.72, 30.65, 30.64, 30.57, 30.55, 30.53, 30.45, 30.19, 30.16, 30.03, 29.99, 29.92, 29.90, 29.1, 29.0, 28.01, 27.97, 26.3, 26.2, 25.7, 25.6, 24.3, 23.77, 23.75, 23.71, 23.67, 23.6, 14.44; HRMS (MALDI-TOF): m/z= 1106.5109, calculated for $C_{56}H_{79}N_7O_{10}S_3$ $(M+H)^+$ 1106.5123; Analytical HPLC (254 nm) t_R 4.429 min, >99% purity.

5-Lys-FAM-RAFT (2)

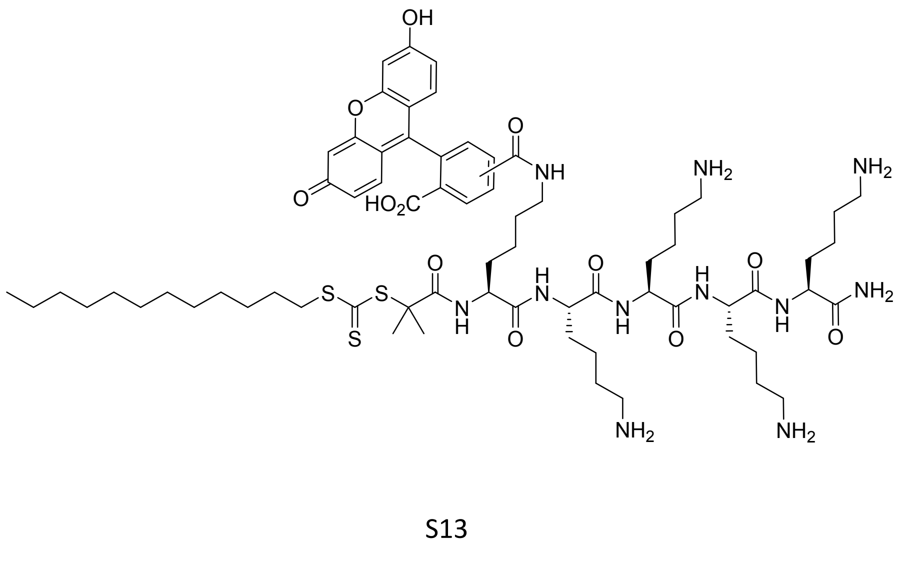

Yield 11% (96 mg, yellow powder). ^{1}H NMR (500 MHz, Methanol-d_4) δ 8.49 – 8.43 (m, 0.5H, *isomer 5*), 8.23 (dd, *J* = 8.0, 1.6 Hz, 0.5H, *isomer 5*), 8.15 (dd, *J* = 8.1, 1.2 Hz, 0.5H, *isomer 6*), 8.12 – 8.05 (m, 0.5H, *isomer 6*), 7.70 – 7.64 (m, 0.5H, *isomer 6*), 7.31 (d, *J* = 8.0 Hz, 0.5H, *isomer 5*), 6.74 – 6.66 (m, 2H), 6.65 – 6.49 (m, 4H), 4.39 – 4.22 (m, 4H), 4.16 (dd, *J* = 8.2, 6.2 Hz, 0.5H, , *isomer 5*), 4.06 (dd, *J* = 9.4, 4.9 Hz, 0.5H, , *isomer 6*), 3.01 – 2.88 (m, 8H), 1.97 – 1.40 (m, 39H), 1.39 – 1.17 (m, 20H), 0.94 – 0.83 (m, 3H); ^{13}C NMR (126 MHz, Methanol-d_4) δ 223.2, 223.1, 176.5, 175.53, 175.45, 174.84, 174.81, 174.24, 174.15, 174.1, 173.97, 173.96, 170.6, 170.5, 168.34, 168.26, 161.6, 154.2, 154.1, 142.3, 137.9, 135.5, 130.4, 130.4, 130.24, 130.17, 128.7, 126.2, 125.8, 125.2, 124.5, 114.0, 113.9, 113.8, 110.98, 110.95, 110.9, 103.7, 57.8, 57.6, 56.6, 56.5, 54.8, 54.74, 54.71, 54.2, 40.49, 40.46, 40.4, 37.8, 37.7, 33.04, 33.03, 32.5, 32.24, 32.18, 32.14, 32.12, 32.04, 31.99, 31.8, 31.6, 30.7, 30.64, 30.63, 30.58, 30.53, 30.52, 30.4, 30.18, 30.15, 30.0, 29.94, 29.91, 29.89, 29.03, 29.01, 27.98, 27.96, 27.9, 26.2, 26.1, 25.7, 25.6, 24.3, 24.2, 23.81, 23.79, 23.75, 23.73, 23.71, 23.70, 23.64, 23.62, 14.4; HRMS (MALDI-TOF) *m/z* 1362.7001 calculated for $C_{68}H_{103}N_{11}O_{12}S_3$ $(M+H)^+$ 1362.7023; Analytical HPLC (254 nm) t_R 3.804 min, >99% purity.

7-Lys-FAM-RAFT (3)

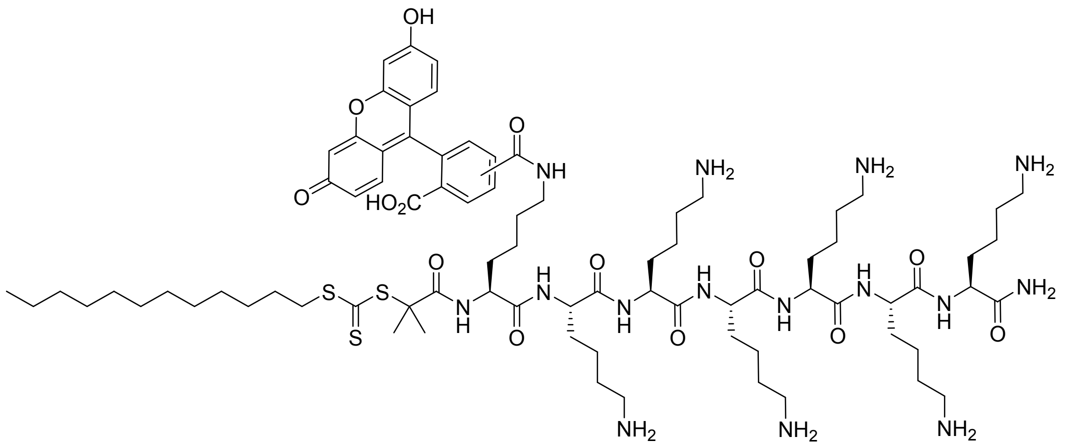

Yield 7% (76 mg, yellow powder). ^{1}H NMR (500 MHz, Methanol-d_4) δ 8.50 – 8.46 (m, 0.5H, *isomer 5*), 8.24 (dd, *J* = 8.1, 1.6 Hz, 0.5H, *isomer 5*), 8.16 (dd, *J* = 8.0, 1.4 Hz, 0.5H, *isomer 6*), 8.12 – 8.06 (m, 0.5H, *isomer 6*), 7.69 – 7.67 (m, 0.5H, *isomer 6*), 7.32 (d, *J* = 8.0 Hz, 0.5H, *isomer 5*), 6.72 – 6.69 (m, 2H), 6.64 – 6.52 (m, 4H), 4.35 – 4.22 (m, 6H), 4.14 (dd, *J* = 9.0, 5.4 Hz, 0.5H, *isomer 5*), 4.04 (dd, *J* = 9.7, 4.7 Hz, 0.5H, *isomer 6*), 3.00 – 2.92 (m, 12H), 2.02 – 1.33 (m, 61H), 1.32 – 1.22 (m, 20H), 0.92 – 0.86 (m, 3H);^{13}C NMR (126 MHz, Methanol-d_4) δ 223.4, 223.3, 176.6, 175.8, 175.7, 175.2, 175.1, 174.5, 174.42, 174.37, 174.1, 170.61, 170.56, 168.34, 168.27, 163.1, 162.8, 161.6, 154.12, 154.07, 142.3, 137.9, 135.6, 130.4, 130.3, 130.3, 130.2, 128.7, 126.2, 125.7, 125.2, 124.5, 119.4, 117.0, 113.9, 113.8, 110.9, 110.9, 103.7, 57.8, 57.7, 57.5, 57.3, 57.1, 57.0, 55.3, 55.2, 55.1, 55.0, 54.9, 54.32, 40.51, 40.48, 40.39, 40.26, 37.87, 37.79, 33.05, 33.04, 32.46, 32.0, 31.91, 31.86, 31.6, 31.4, 30.72, 30.71, 30.68, 30.64, 30.55, 30.53, 30.4, 30.20, 30.17, 30.0, 29.92, 29.90, 29.04, 29.01, 28.00, 27.97, 27.9, 26.3, 26.1, 25.72, 25.66, 24.3, 24.2, 23.9, 23.8, 23.7, 17.6, 17.4, 17.3, 17.1, 17.0, 14.4; HRMS (MALDI-TOF) *m/z* 1618.8881, calculated for $C_{80}H_{127}N_{15}O_{14}S_3$ $(M+H)^+$ 1618.8922; Analytical HPLC (254 nm) t_R 3.496 min, >99% purity.

2.3. Synthesis of Control RAFT agent 4

1-((2-aminoethyl)amino)-2-methyl-1-oxopropan-2-yl dodecyl carbonotrithioate hydrochloride (5)

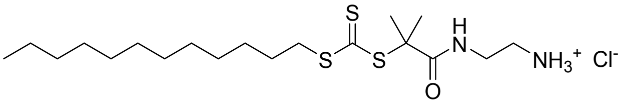

DDMAT (100 mg, 0.27 mmol, 1 equiv.) and Oxyma (45 mg, 0.32 mmol, 1.2 equiv.) were dissolved in DMF (4 mL) and stirred for 10 min. EDC (61 mg, 0.32 mmol, 1.2 equiv.) was added and the mixture was stirred for 1 min. *N*-Boc-1,2-diaminoethane (62 mg, 61.7 µL, 0.39 mmol, 1.4 equiv.) in DMF (1 mL) was slowly added and the reaction mixture was stirred at 45°C for 4 h. The mixture was then concentrated under reduced pressure. The residue was dissolved in Et_2O (20 mL), then washed with an aqueous solution of $NaHCO_3$ (3 × 15 mL) and with H_2O. The organic layer was dried with $MgSO_4$, filtered and evaporated *in vacuo*. The Boc group was directly removed by dissolving the product with a solution of HCl in Et_2O (2M, 4mL) and stirring for 24 h. The resulting precipitate was collected by filtration, washed with Et_2O, and air dried to give **5** as a light-yellow solid.

Yield 78% (85 mg, light yellow powder). ^1H NMR (500 MHz, Methanol-d_4) δ 3.42 (t, *J* = 6.4 Hz, 2H), 3.33 (t, *J* = 7.4 Hz, 2H), 3.03 (t, J = 6.3 Hz, 2H), 1.68 (s, 7H), 1.72 – 1.62 (m, 2H), 1.45 – 1.22 (m, 18H), 0.90 (t, *J* = 6.9 Hz, 3H).; ^{13}C NMR (126 MHz, Methanol-d_4) δ 223.7, 176.6, 57.8, 40.6, 39.1, 37.7, 33.1, 30.73, 30.65, 30.6, 30.5, 30.2, 29.9, 29.1, 25.9, 23.7, 14.4. LCMS (ESI) *m/z* 407.2; Analytical HPLC (220 nm) t_R 5.237 min, >99% purity.

0-Lys-FAM RAFT (4)

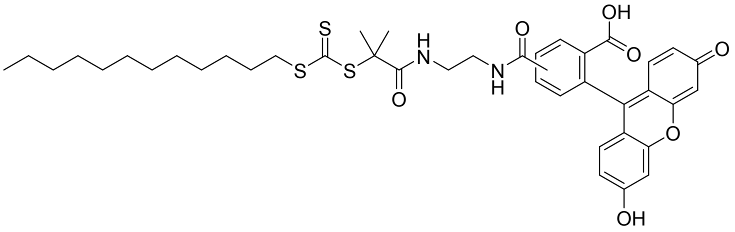

5,6-carboxyfluorescein (100 mg, 0.266 mmol, 1.1 equiv.) and Oxyma (45 mg, 0.32 mmol, 1.2 equiv.) were dissolved in DMF (4 mL) and stirred for 10 min. EDC (65 mg, 0.32 mmol, 1.2 equiv.) was added and the mixture was stirred for 1 min. A solution of **6** (90 mg, 0.22 mmol, 1 equiv.) and DIPEA (76.6 µL, 0.44 mmol, 2 equiv.) in DMF (1 mL) (previously stirred for 15 min) was slowly added and the reaction mixture was stirred at 45 °C for 4 h (monitored by TLC, eluting with DCM:MeOH, 9/1). The mixture was filtered then evaporated under reduced pressure. The residue was dissolved in EtOAc (20 mL), and washed with H_2O (6 x 10 mL). The organic layer was dried with $MgSO_4$, filtered and dried *in vacuo*. The crude product was purified by flash column chromatography (gradient eluent: DCM/MeOH, 99:1 to 80:20), yielding **4** as a yellow solid.

Yield 18% (orange powder, 30 mg). ^1H NMR (500 MHz, Methanol-d_4) δ 8.44 – 8.39 (m, 0.5H, *isomer 5*), 8.18 (dd, *J* = 8.0, 1.6 Hz, 0.5H, *isomer 5*), 8.12 (dd, *J* = 8.0, 1.4 Hz, 0.5H, *isomer 6*), 8.10 – 8.05 (m, 0.5H, *isomer 6*), 7.62 (d, *J* = 1.3 Hz, 0.5H, *isomer 6*), 7.29 (d, *J* = 8.0 Hz, 0.5H, *isomer 5*), 6.72 – 6.67 (m, 2H), 6.61 (t, *J* = 8.8 Hz, 2H), 6.54 (dt, *J* = 8.7, 2.5 Hz, 2H), 3.59 – 3.52 (m, 1H, *isomer 5*), 3.49 – 3.43 (m, 1H,

isomer 5), 3.43 – 3.39 (m, 1H, *isomer 6*), 3.37 – 3.33 (m, 1H, *isomer 6*), 3.19 – 3.13 (m, 1H, *isomer 5*), 3.13 – 3.06 (m, 1H, *isomer 6*), 1.68 (s, 3H, *isomer 5*), 1.58 – 1.50 (m, 2H), 1.54 (s, 3H, *isomer 6*), 1.33 – 1.21 (m, 18H), 0.94 – 0.87 (m, 3H);^{13}C NMR (126 MHz, Methanol-d_4) δ 223.0, 222.7, 175.9, 175.71, 175.68, 175.2, 173.0, 170.6, 168.7, 168.4, 162.2, 154.3, 154.2, 141.8, 140.2, 139.3, 137.6, 135.7, 135.3, 130.9, 130.42, 130.35, 130.2, 127.09, 127.05, 126.4, 125.9, 125.5, 125.2, 124.5, 116.5, 114.2, 111.2, 111.0, 103.7, 57.9, 57.7, 41.3, 40.9, 40.8, 37.7, 37.6, 33.1, 30.9, 30.7, 30.63, 30.62, 30.51, 30.45, 30.4, 30.14, 30.09, 29.93, 29.88, 29.8, 29.7, 29.6, 29.03, 28.99, 26.10, 26.07, 26.0, 23.7, 18.4, 17.4, 17.3, 17.1, 14.5. HRMS (MALDI-TOF) *m/z* 765.2721, calculated for $C_{40}H_{48}N_2O_7S_3$ $(M+H)^+$ 765.2696. Analytical HPLC (254 nm) t_R 6.177 min, 95% purity.

2.4. Polymerization reactions

Fluorescently-labelled PDMA polymers were synthesized using RAFT agents **1**–**4** with a theoretical yield of ~50 mg of the resulting polymer. See Table **S1** for amount of monomer, RAFT agent, and the initiator AIBN used for each polymer.

Table S2. Quantities of starting material used for the polymerization reactions.

Polymer	monomer (µL)	RAFT agent (mg)	AIBN (µg)
**3Lys-PDMA-7.5k**	54	7	23
**5Lys-PDMA-7.5k**	51	9	22
**7Lys –PDMA-7.5k**	48	10	20
**0Lys-PDMA-7.5k**	49	5.5	21
**3Lys-PDMA-50k**	51	1.0	33
**5Lys-PDMA-50k**	53	1.4	34
**7Lys-PDMA-50k**	51	1.6	32
**0Lys-PDMA-50k**	52	0.8	34
**3Lys-P(2HEA)-50k**	52	1.0	28
**3Lys-PS-50k**	52	1.1	31

Polymerization of DMA with RAFT agents 1–**3**. DMA:RAFT:AIBN molar ratios of 75:1:0.02 and 500:1:0.02 were used to prepare the 7.5 kDa Mw and 50 kDa polymers, respectively. The final polymerization concentrations of the monomers were ~0.70 M. Polymerizations were performed in a mixture of D_2O:1,4-dioxane (10:90) in a sealed tube equipped with a stirring bar. The reaction mixture was first degassed by bubbling argon for 30 min and then placed into a 70 °C oil bath for 4 h. The polymerizations were quenched with liquid nitrogen once maximum monomer conversion had been achieved (monitored using ^{1}H NMR). After defrosting in air, D_2O was added to dissolve the precipitated PDMA polymer and final monomer conversion was determined by ^{1}H NMR. Polymers were then dried *in vacuo,* precipitated in Et_2O, and consecutively dissolved in DMF (× 1) and MeOH (× 2) with precipitation in Et_2O. Finally, polymers were dialyzed in water for 24 h using 1 kDa and 15 kDa molecular weight cut off dialysis tubing (Spectrum™, Spectra/Por™) for the 7.5 kDa and 50 kDA polymers, respectively. Polymers were isolated after lyophilizing to dryness.

Polymerization of 2HEA with RAFT agent 1. A 2HEA:RAFT:AIBN molar ratios of 500:1:0.02 was used to prepare a 50 kDa polymer. The final polymerization concentration of the monomer was ~0.70 M. The polymerization was performed in a mixture of D_2O:1,4-dioxane (10:90) in a sealed tube equipped with a stirring bar. The reaction mixture was first degassed by bubbling argon for 30 min and then placed into a 70 °C oil bath for 4 h. The polymerization was quenched with liquid nitrogen once maximum monomer conversion had been achieved (monitored using ^{1}H NMR). The polymer was then precipitated in hexane, and consecutively washed with Et_2O.

Polymerization of styrene with RAFT agent 1. A styrene:RAFT:AIBN molar ratios of 500:1:0.02 was used to prepare a 50 kDa polymer. The final polymerization concentration of the monomer was ~0.70 M. The polymerization was performed in a mixture of D_2O:1,4-dioxane (10:90) in a sealed tube equipped with a stirring bar. The reaction mixture was first degassed by bubbling argon for 30 min and then placed into a 70 °C oil bath for 48 h. The polymerization was quenched with liquid nitrogen once monomer conversion was satisfactory (monitored using ^{1}H NMR). The polymer was then precipitated in MeOH, and consecutively washed with Et_2O.

Polymerization of DMA with RAFT agent 4. The polymerizations were performed under the same conditions as above but at 65 °C, and the reaction was quenched after 6 h (~95 % conversion, as determined by ^{1}H NMR).

2.5. Polymer characterization

^{1}H NMR and GPC

Polymer length of the PDMA chains was determined by integrating the two methyl groups on the repeating units of the PDMA (6H, N(CH_3)$_2$ at 2.85–3.10 ppm) relative to the terminal methyl group (3H, CH_3, 0.92 ppm).

Polymer length of the P(2HEA) chains was determined by integrating the two signals corresponding to the repeating units of the P(2HEA) backbone (3H, CH at 2.67 – 2.27 ppm and CH_2 at 2.05 – 1.55 ppm) relative to the terminal methyl group (3H, CH_3, 0.91 ppm).

Polymer length of the PS chains was determined by integrating the 5 protons on the aryle side chain of the PS repeating units (5H, at 7.37 – 6.31 ppm) relative to the terminal methyl group (3H, CH_3, 0.98 ppm).

GPC samples were prepared in solutions of DMF containing 0.1 % w/v LiBr at concentrations of 5 mg/ml of polymer. Samples were run at 60 °C for 30 minutes at a flow rate of 1 mL/min.

Fluorescence Characterization.

Stock solutions (2mg/mL) of the polymers in Dulbecco's PBS were prepared and sequentially diluted and plated into a black Corning Costar Assay 96 well plate. ~7.5 kDa polymer samples were prepared

with a concentration range of 0–200 μg/mL and ~50 kDa polymer samples with a range of 0–1000 μg/mL. A BioTek HT Synergy multi-mode reader was used to determine sample fluorescence. Samples were excited at 480 nm and fluorescence emission was recorded at 520 nm.

Critical micelle concentration assays

To assess any potential self-assembly behavior of our polymers, critical micelle concentration (CMC) measurements were performed using a Nile Red encapsulation assay adapted from the literature.[2] A solution of Nile Red (100 μM, 15 μL) in CH_2Cl_2 was added into separate glass vials (1.5 mL), and the solutions were allowed to dry in air. Solutions of the PDMA polymers (4.7 – 75 mg/mL) in H_2O were added (500 μL), and the mixture stirred in the dark overnight. An aliquot (100 μL) of the resulting solution was transferred into an Ultra-Micro quartz cuvette (Hellma 105.250-QS), and the fluorescence of the samples (λ_{ex} = 520 nm) was recorded, and the fluorescence intensity of the samples at 655 nm was plotted as a function of concentration, as shown in Figure S12. The fluorescence of Nile Red, which is poorly soluble in water but fluorescent in a hydrophobic environment, should increase when amphiphilic polymers self-assemble into micelles, leading to dye encapsulation. The concentration at which the fluorescence of the samples deviates from linearity and begins to increase is therefore the CMC. Here, no fluorescence increase was observed, indicating no self-assembling behavior on this concentration range.

2.6. Biological Assays

Cell Culture

HeLa and MCF7 cells were cultured in Dulbecco's modified Eagle medium (DMEM) supplemented with L-glutamine (4 mM), 10 % fetal bovine serum (FBS), and antibiotics (penicillin and streptomycin, 100 units/mL). Cell culture was performed in a SteriCult 200 (Hucoa-Erloss) incubator at 5 % CO_2 atmosphere at 37 °C. To culture and plate the cells, the cells were washed with PBS, detached with trypsin/EDTA (0.25 % trypsin, 1 mM in PBS), diluted in DMEM, counted, then further diluted with DMEM to the appropriate concentration.

Cell Viability Assay

HeLa and MCF7 cells were plated in a 96-well plate at 5,000 cells per well and allowed to grow to ~70% confluency overnight. Cells were incubated for 24 h with solutions at desired concentrations of **0Lys-PDMA-7.5k**, **3Lys-PDMA-7.5k**, **5Lys-PDMA-7.5k**, **7Lys-PDMA-7.5k** (5, 50, and 400 μg/mL), or **0Lys-PDMA-50k**, **3Lys-PDMA-50k**, **5Lys-PDMA-50k**, **7Lys-PDMA-50k** (5, 50, 400, and 1000 μg/mL). The media was replaced with 100 μL of 3-(4,5-dimethylthiazol-2-yl)-2,5-diphenyltetrazolium bromide (MTT) solution (1 mg/mL) in PBS and the cells incubated for a further 3 h at 37 °C. After incubation, the resulting formazan crystals were dissolved by adding 100 μL of MTT solubilization solution (10 % Triton-X 100 in 0.1 N HCl in isopropanol). The absorbance was measured at a wavelength of 570 nm (BioTek HT Synergy multi-mode reader) and the results compared to untreated (control) cells.

Cellular Uptake Studies

HeLa and MCF7 cells were plated on a 24-wellplate at a density of 2 × 10^4 cells/well and grown to 70% confluency overnight at 37°C. Stock solutions of polymers **0Lys-PDMA-7.5k**, **3Lys-PDMA-7.5k**, **5Lys-PDMA-7.5k**, **7Lys-PDMA-7.5k**, or **0Lys-PDMA-50k**, **3Lys-PDMA-50k**, **5Lys-PDMA-50k**, **7Lys-PDMA-50k** were prepared (2 mg/mL in PBS). Samples were added into the wells (volumes were calculated to normalize the brightness with respect to the 5lys polymers, using the fluorescence data reported on Figure S3) and diluted to 50 µg/mL by adding complete DMEM media (500 µL). The cells were then incubated for 24 h. After incubation, the cells were washed twice with PBS and harvested with 500 µL trypsin/EDTA (0.25 % trypsin, 1 mM in PBS), transferred to falcon tubes, and the trypsin/EDTA deactivated by adding fresh 500 µL complete DMEM. The cells were washed twice via centrifugation with complete DMEM and resuspended in FACS buffer (PBS, 10% FBS, 0.1% NaN_3) and analyzed by flow cytometry.

Live Cell Confocal Microscopy

HeLa cells were plated on an 8-well µ-Slide (ibidi GmbH, Germany) with 10 × 10^4 cells/well and grown to 40 % confluency for 24 h at 37 °C. The media was removed and replaced with ~7.5 kDa polymer solutions in DMEM (200 µg/mL), and CellLight® early Endosomes-RFP, BacMam 2.0 (ThermoFisher) solution was added according to the manufacturer's instructions, and the cells incubated for 24 h at 37 °C. After incubation, cells were washed twice with media, stained with Hoechst 33342 for 20 min and imaged in supplemented FluoroBrite™ DMEM with a Leica SP5 confocal microscope. Microscope lasers settings were: excitation laser lines at 405 nm, 488 nm, and 595 nm with emission filters of 385–470 nm for Hoechst 33342 (nuclei stain), 505–530 nm for FAM labelled polymers, and 595–615 nm for RFP (CellLight® early Endosomes-RFP).

3. NMR, HRMS (MALDI), and HPLC characterizations

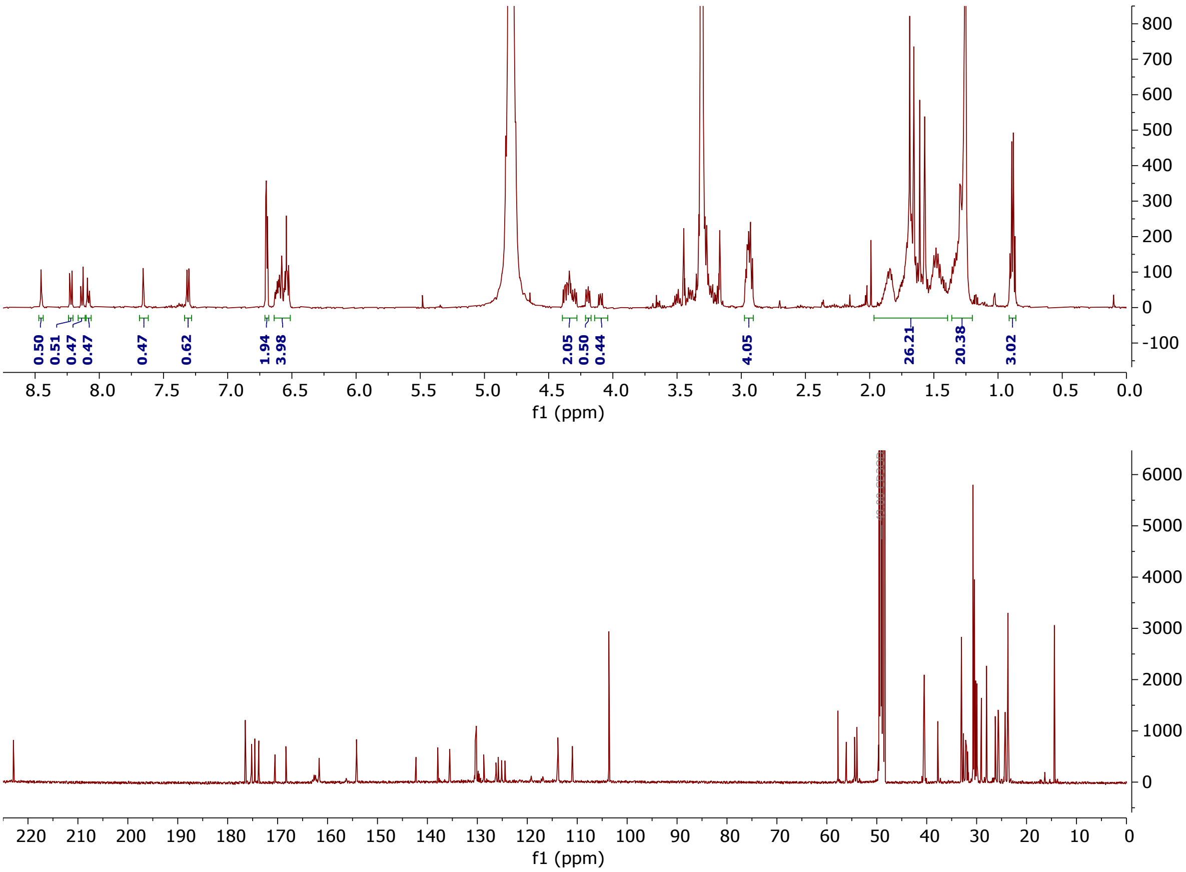

Figure S13. ^{1}H and ^{13}C NMR spectra of compound **1** recorded in Methanol-d_4 at 500 MHz and 126 MHz, respectively

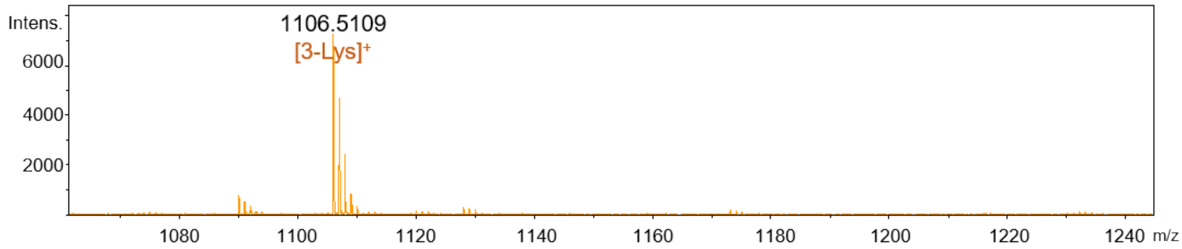

Figure S14. HRMS (MALDI) spectrum for compound **1**

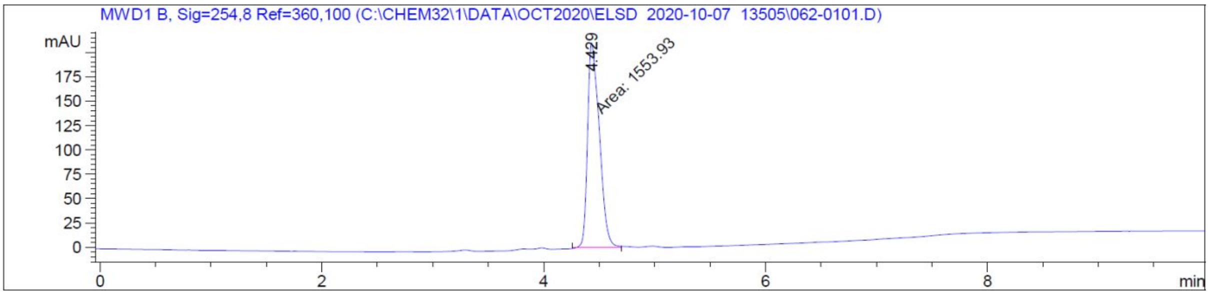

Figure S15. HPLC trace for compound **1**

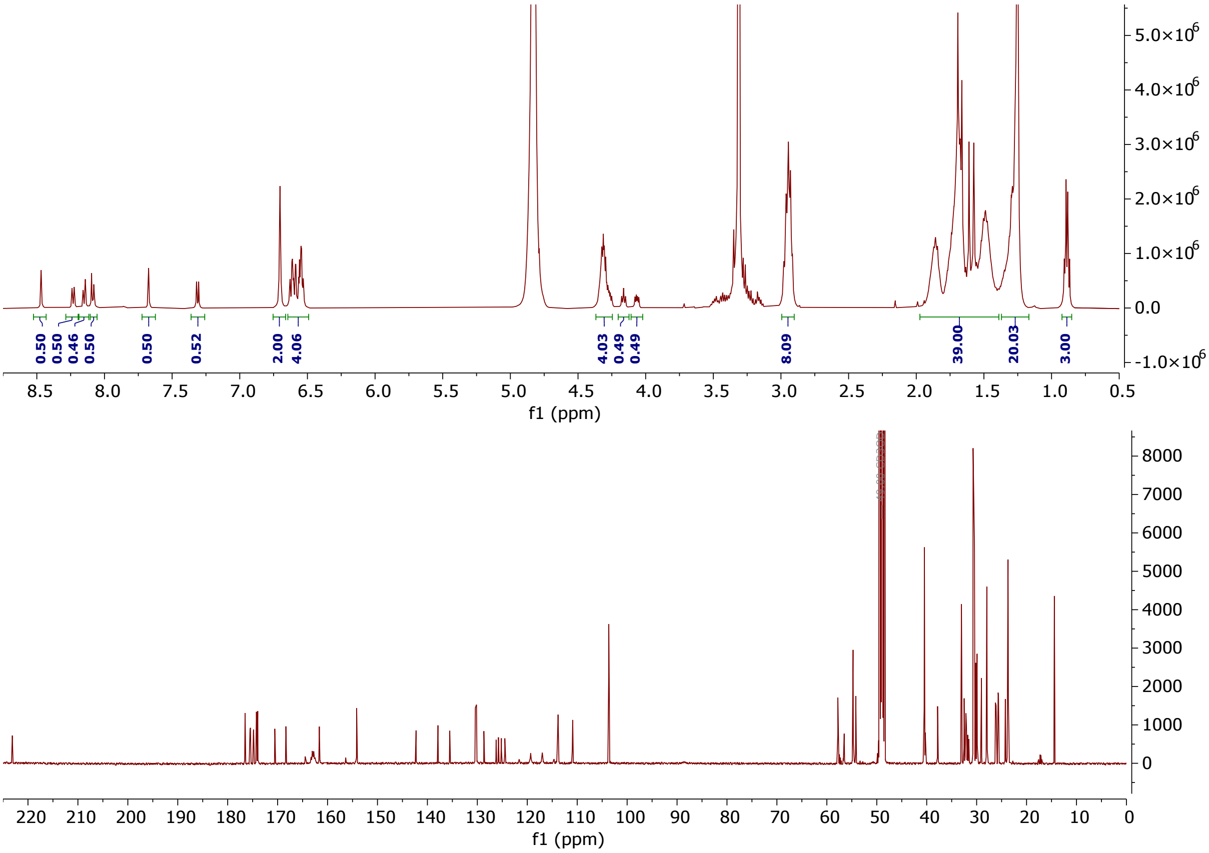

Figure S16. ^{1}H and ^{13}C NMR spectra of compound **2** recorded in Methanol-d_4 at 500 MHz and 126 MHz, respectively

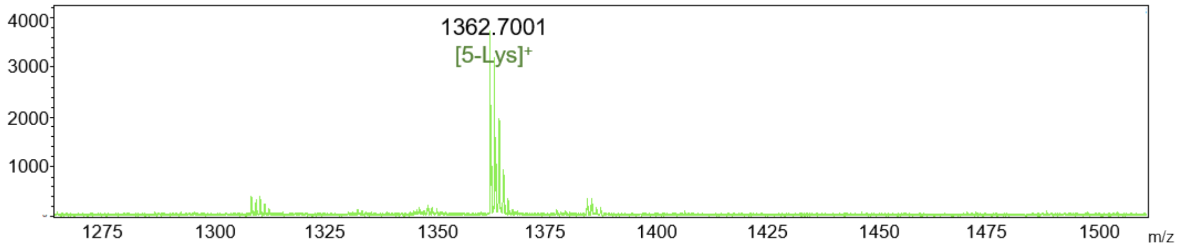

Figure S17. HRMS (MALDI) spectrum for compound **2**

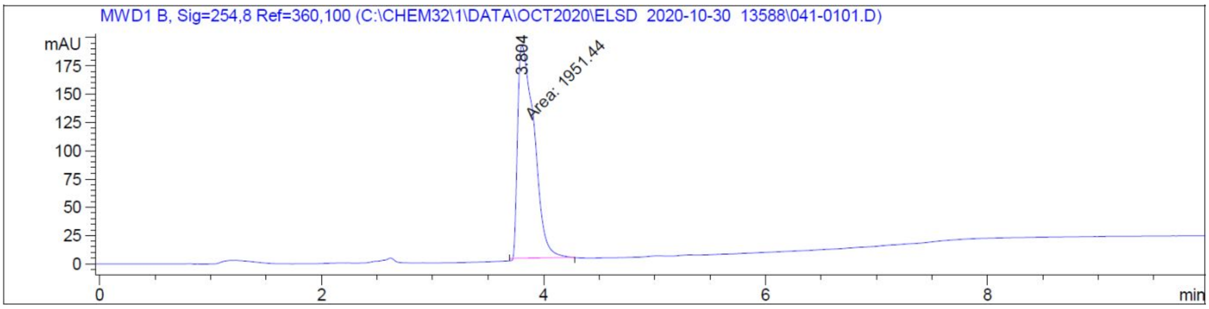

Figure S18. HPLC trace for compound **2**

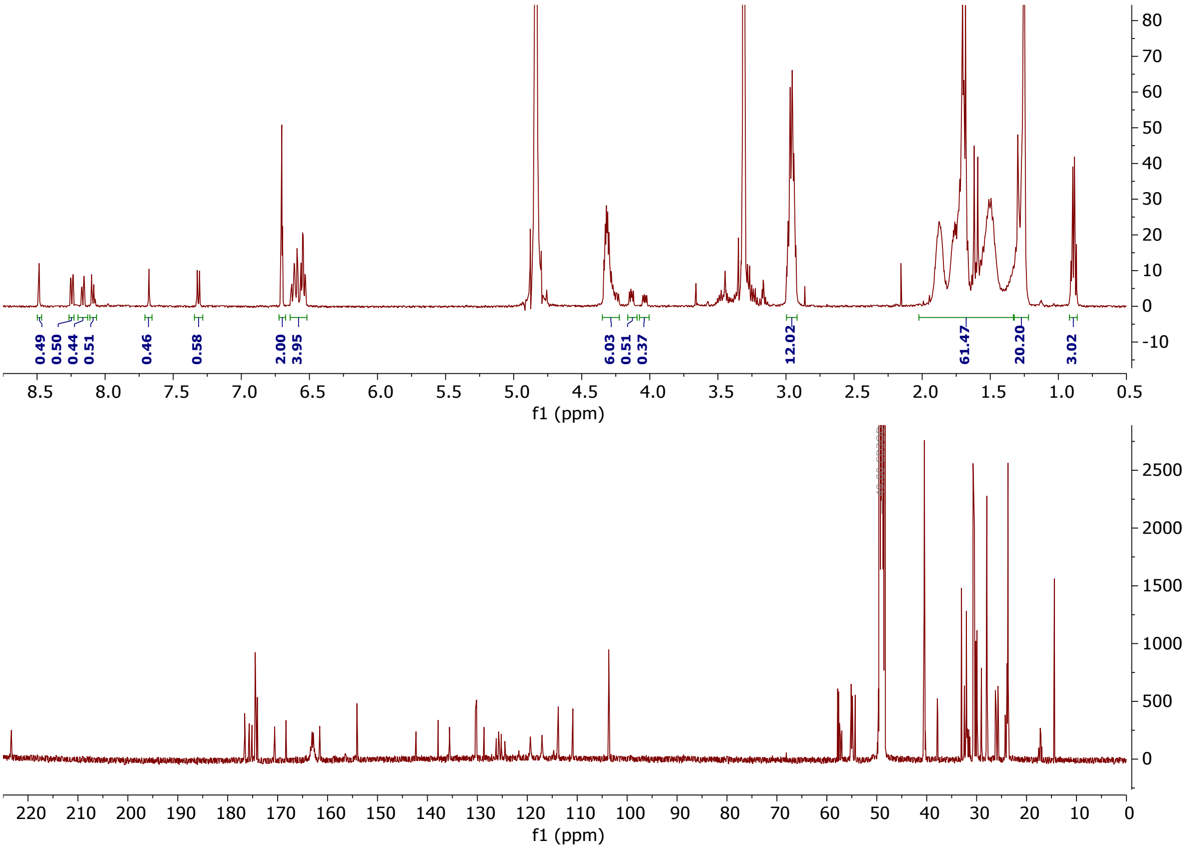

Figure S19. ^{1}H and ^{13}C NMR spectra of compound **3** recorded in Methanol-d_4 at 500 MHz and 126 MHz, respectively.

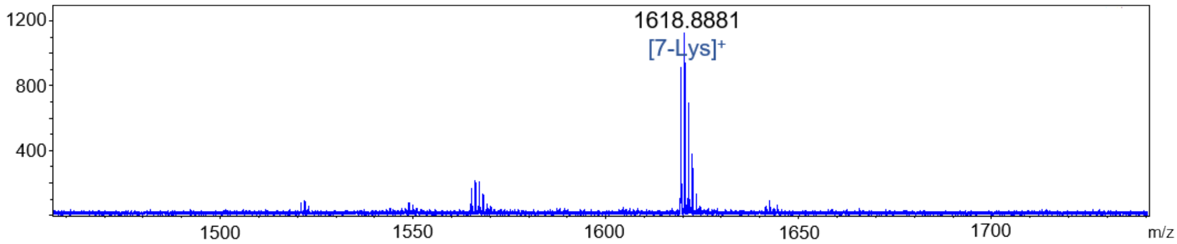

Figure S20. HRMS (MALDI) spectrum for compound **3**

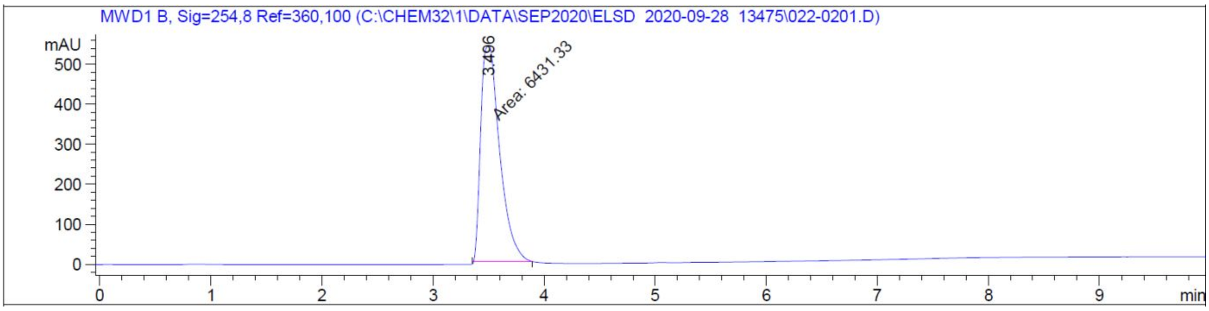

Figure S21. HPLC trace for compound **3**

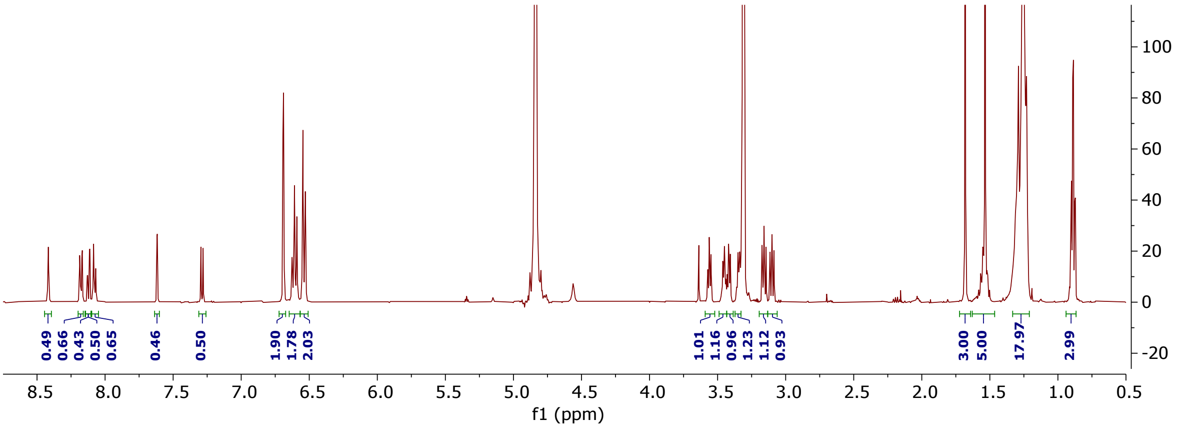

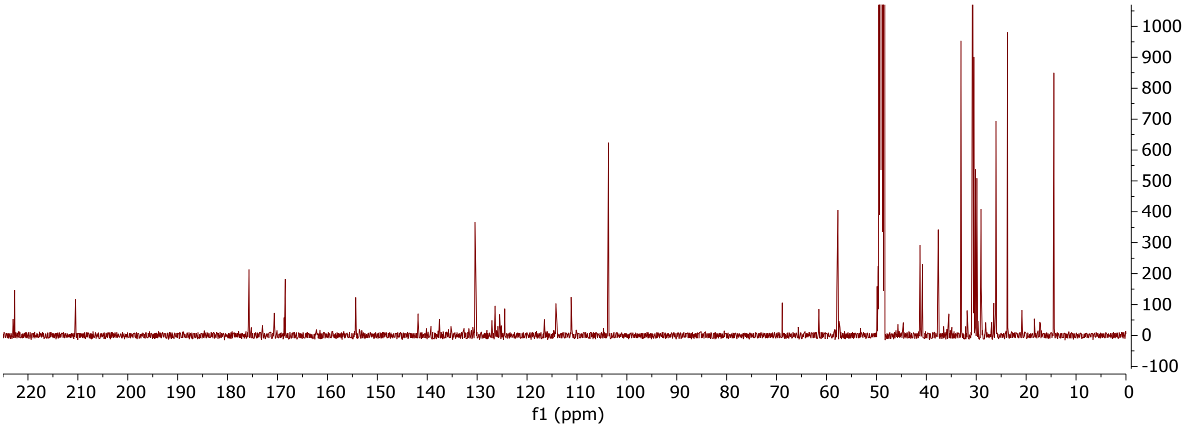

Figure S22. ^{1}H and ^{13}C NMR spectra of compound **4** recorded in Methanol-d_4 at 500 MHz and 126 MHz, respectively.

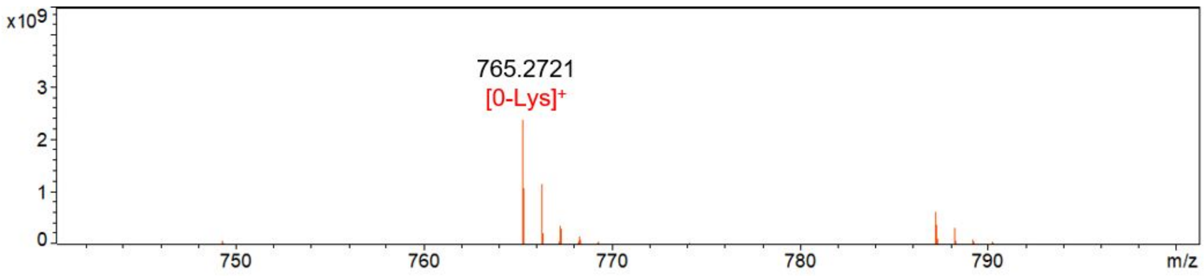

Figure S23. HRMS (MALDI) spectrum for compound **4**

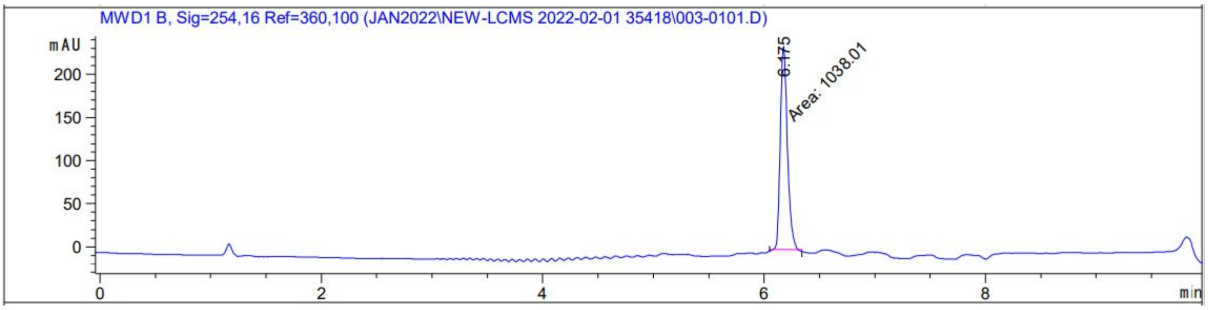

Figure S24. HPLC trace for compound **4**

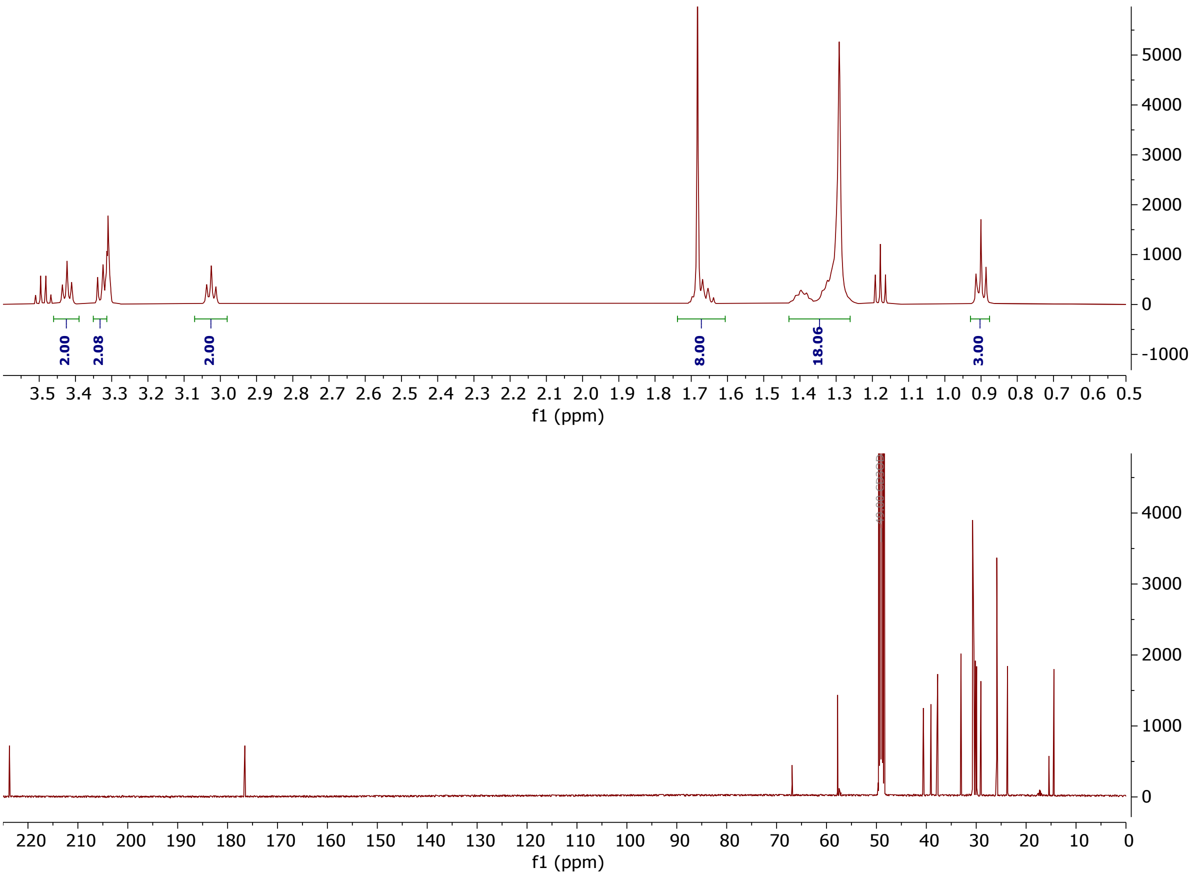

Figure S25. ^{1}H and ^{13}C NMR spectra of compound **5** recorded in Methanol-d_4 at 500 MHz and 126 MHz, respectively.

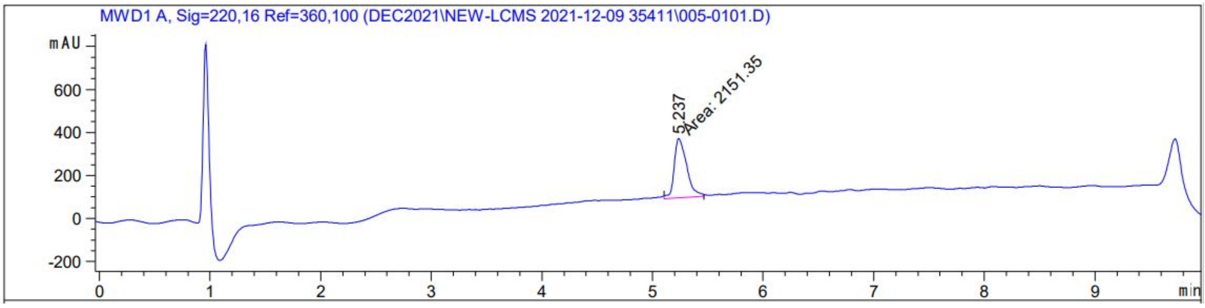

Figure S26. HPLC trace for compound **5**

4. Supplementary references

(1) Díaz-Mochón, J. J.; Bialy, L.; Watson, J.; Sánchez-Martín, R. M.; Bradley, M. Synthesis and cellular uptake of cell delivering PNA–peptide conjugates. *Chemical Communications* **2005**, (26), 3316-3318, 10.1039/B503777H. DOI: 10.1039/B503777H.
(2) Dariva, C. G.; Figueiredo, J. P. H.; Ferreira, C.; Laranjo, M.; Botelho, M. F.; Fonseca, A. C.; Coelho, J. F. J.; Serra, A. C. Development of red-light cleavable PEG-PLA nanoparticles as delivery systems for cancer therapy. *Colloids and Surfaces B: Biointerfaces* **2020**, *196*, 111354. DOI: https://doi.org/10.1016/j.colsurfb.2020.111354.
